# Advances in the Hydroperoxidation of Propylene to Propylene Oxide (HOPO): from *Nanoscale* to *Mesoscale* and *Macroscale*

**DOI:** 10.1002/chem.202501205

**Published:** 2025-08-25

**Authors:** Nidhi Kapil, Marc‐Olivier Coppens

**Affiliations:** ^1^ Centre for Nature‐Inspired Engineering and Department of Chemical Engineering University College London London WC1E 7JE UK

**Keywords:** bimetallic nanoparticles, catalyst, gold nanoparticles, propylene epoxidation, supports

## Abstract

Hydroperoxidation of propylene to propylene oxide (HOPO) using H_2_ and O_2_ offers several advantages over commercial methods such as the chlorohydrin process and the hydroperoxide process. This review presents a comprehensive exploration of the advancements in the HOPO, with a focus on catalyst development and reactor engineering. Various catalyst synthesis strategies, including the modulation of gold nanoparticle (AuNP) size, role, and types of support, and the potential of bimetallic nanoparticles are discussed. The catalytic properties of nickel and the reaction mechanism involved in the epoxidation are also presented. These strategies offer promising pathways to enhance catalyst stability, selectivity, and overall performance. Additionally, this article highlights the critical role of reactor engineering, showcasing the significance of different reactor configurations and feed concentrations enabling higher reactant concentrations that avert explosions. The review identifies key challenges and opportunities across different scales–nanoscale, mesoscale, and macroscale – with the aim to provide valuable insights and guidance for future advancements in the field of propylene epoxidation.

## Graphical abstract

This review highlights advancements in HOPO across *nanoscale*, *mesoscale*, and *macroscale*. It explores key aspects such as nanoparticle design, support materials, catalyst preparation methodologies, and reactor engineering. Additionally, the review highlights the impact of bimetallic nanoparticles, reaction mechanisms, and process optimizations that have contributed to improving catalyst stability, selectivity, and overall efficiency.

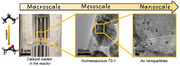

## 1 Introduction

Propylene oxide (PO) is an important chemical that holds substantial significance as a chemical building block, and is used in the production of many commercial materials.^[^ ^1^ ^]^ One of the primary applications of PO is in the synthesis of polyurethanes, which are subsequently used in the production of foams for mattresses, cushions, automobiles, food packaging, and insulation materials.^[^ ^2^, ^3^, ^4^ ^]^ It also acts as an intermediate for the production of chemicals like propylene glycols, which are essential in cosmetics, pharmaceuticals, and antifreeze formulations.^[^ ^2^, ^5^ ^]^ PO is also used to synthesize propylene glycol ethers and acetates, used as a solvent in paintings, coatings, and printing industries.^[^ ^6^ ^]^ Furthermore, PO is used in producing speciality chemicals, like propylene carbonate (a battery electrolyte), isopropanolamines (in detergents, adhesives), and allyl alcohols (in glycerol synthesis).^[^ ^7^ ^]^ Therefore, the annual demand of PO is increasing remarkably world‐wide and is expected to surpass 20 million tons by the year 2030.^[^ ^8^ ^]^

Various industrial processes, such as the chlorohydrin process and hydroperoxide process, are used for the large‐scale production of PO.^[^ ^2^, ^9^ ^]^ However, these methods are considered environmentally unfriendly, energy intensive, and yielding undesirable co‐products, prompting exploration of more sustainable alternatives.^[^ ^10^ ^]^ Direct gas phase epoxidation using H_2_ and O_2_, also known as hydroperoxidation of propylene to propylene oxide (HOPO), has attracted considerable research interest, because it is a simple, greener, one‐step, and sustainable process; however, it has yet to see industrial implementation due to various challenges, such as insufficient productivity, and lack of a robust catalyst.^[^ ^11^, ^12^, ^13^ ^]^ This reaction is mainly carried out in the presence of gold‐supported titanium(IV) (Au/Ti(IV)) containing catalyst. Small gold nanoparticles (AuNPs) in close proximity with isolated tetrahedral titanium (Ti) sites are necessary for in situ generation of peroxo species, which further react with propylene to form PO.^[^ ^14^, ^15^, ^16^, ^17^ ^]^ Therefore, designing a stable, efficient, yet selective catalyst is crucial for this reaction. Several factors play an important role in the catalytic performance, such as AuNP size, metal loading, methods of preparation, support structure, crystallinity, additives, and promoters.^[^ ^18^, ^19^, ^20^, ^21^, ^22^ ^]^ This has led to extensive research exploring the utilization of various catalysts and reaction methodologies to achieve enhanced PO production rates, H_2_ efficiency (how effectively the H_2_ is used in the formation of PO without being wasted in the formation of water) and PO selectivity.^[^ ^21^, ^23^ ^]^ Along with the development of the catalyst, reactor engineering is equally important to translate the advantages of the catalyst to the production scale.^[^ ^24^ ^]^ Therefore, a multiscale approach, integrating nano‐ (active site), meso‐ (porous catalyst architecture), and macroscale (reactor) efforts, is essential to improve the HOPO method.^[^ ^25^, ^26^ ^]^

This review provides a comprehensive and **integrated multiscale perspective** on the HOPO, covering developments across the *nano‐, meso‐*, and *macroscale* aspects, which are rarely addressed together. It aims to bridge fundamental research in catalyst design, support materials, and reactor technologies with process‐level considerations that are essential for industrial application. The key technological and economic parameters discussed in this review are summarized in Table 1.

**Table 1:** Technological and economic parameters considered in this review.

Parameter	Calculations
H_2_ efficiency/utilization (%)	$H_{2}\;\mathrm{efficiency}=\frac{F_{\mathrm{PO}}^{\mathrm{out}}}{F_{H2}^{\mathrm{in}}\;-\;F_{H2}^{\mathrm{out}}}\times 100$
Propylene Conversion (%)	$\mathrm{Propyleneconversion}=\frac{\frac{1}{3}\;F_{\mathrm{COx}}^{\mathrm{out}}\;+\;\frac{2}{3}\;F_{\mathrm{ethanal}}^{\mathrm{out}}\;+\;\sum F_{C3_{\mathrm{oxygenates}}}^{\mathrm{out}}}{F_{C36H}^{\mathrm{in}}}\times 100$
Propylene Selectivity (%)	$\mathrm{Propylene}\;\mathrm{selectivity}=\frac{F_{\mathrm{PO}}^{\mathrm{out}}}{\frac{1}{3}\;F_{\mathrm{COx}}^{\mathrm{out}}\;+\;\frac{2}{3}\;F_{\mathrm{ethanal}}^{\mathrm{out}}\;+\;\sum F_{C3_{\mathrm{oxygenates}}}^{\mathrm{out}}}\times 100$
PO production rate (g_PO_ h^−1^ kg^−1^_cat_)	$\mathrm{PO}\;\mathrm{prod}\mathrm{ucti}\mathrm{on}\;\mathrm{rate}=\frac{F_{\mathrm{PO}}^{\mathrm{out}}}{\mathrm{cata}\mathrm{lyst}\;\mathrm{mass}}\times M_{\mathrm{PO}}\times 60$

Where *F*_i_ is the molar flow rate of the generic i^th^ species, in mol minute^−1^, catalyst mass is in kg and *M*_PO_ is the molar mass of PO in g/mol.

On the *nanoscale*, the synthesis of AuNPs, influence of the AuNP size, use of various bimetallic nanoparticles, and their different preparation methodologies are discussed. Different types of stabilizing ligands are employed to tune the electronic and geometric properties of the nanoparticle's surface, while additives and promoters (like alkaline metals) manipulate the interfacial sites between metal nanoparticles and their supports.^[^ ^27^, ^28^, ^29^ ^]^ AuNPs of size less than 5 nm are found to be catalytically active for this reaction.^[^ ^30^ ^]^ Beyond AuNPs, the catalytic efficacy of nonnoble elements, such as nickel, is also presented. Transitioning to the *mesoscale*, different Ti‐containing supports including TiO_2_, titanium silicalite (TS‐1), mesoporous silica, and core‐shell TS‐1 are discussed along with strategies to modulate their structural attributes and mass‐transfer abilities. AuNPs are deposited onto these supports using different immobilisation techniques. These methods can influence parameters such as the size of AuNPs, loading, dispersion, and efficacy, consequently affecting the overall catalyst performance.^[^ ^31^, ^32^, ^33^ ^]^

Furthermore, the impact of the support's crystal planes, crystal size, Ti loading, pore size, calcination temperature, and pretreatments on catalyst performance is also discussed. Various approaches utilized to refine catalyst design while ensuring long‐term stability, and countering challenges such as nanoparticle aggregation and catalyst deactivation are thoroughly reviewed.^[^ ^34^ ^]^ Catalyst deactivation mainly occurs when the active sites are blocked by unwanted carbonaceous species or due to sintering of NPs.^[^ ^35^ ^]^ Improving the catalyst lifetime is an important parameter for an economically viable and sustainable industrial process. Different strategies are examined to extend catalyst lifespan, such as ex situ or in situ regeneration, safeguarding nanoparticles through capping agents, promoters, or encapsulation.^[^ ^36^, ^37^, ^38^ ^]^ On the *macroscale*, distinct types of epoxidation reactors, such as a conventional packed bed reactor, membrane reactor, and microreactor are described. Various facets, such as reaction feed concentration, reactor configuration, feeding strategies, reaction temperature, and pressure are explored in the context of refining the propylene epoxidation process. Using a microreactor or membrane reactor enabled the investigation of reaction kinetics within the explosive regime. The reaction mechanism studies by DFT (density functional theory) calculation, CFD (computational fluid dynamics) techniques, and advanced in situ spectroscopy are thoroughly examined in this review.

## 2 Industrial Processes to Produce Propylene Oxide (PO)

**Figure 1:**
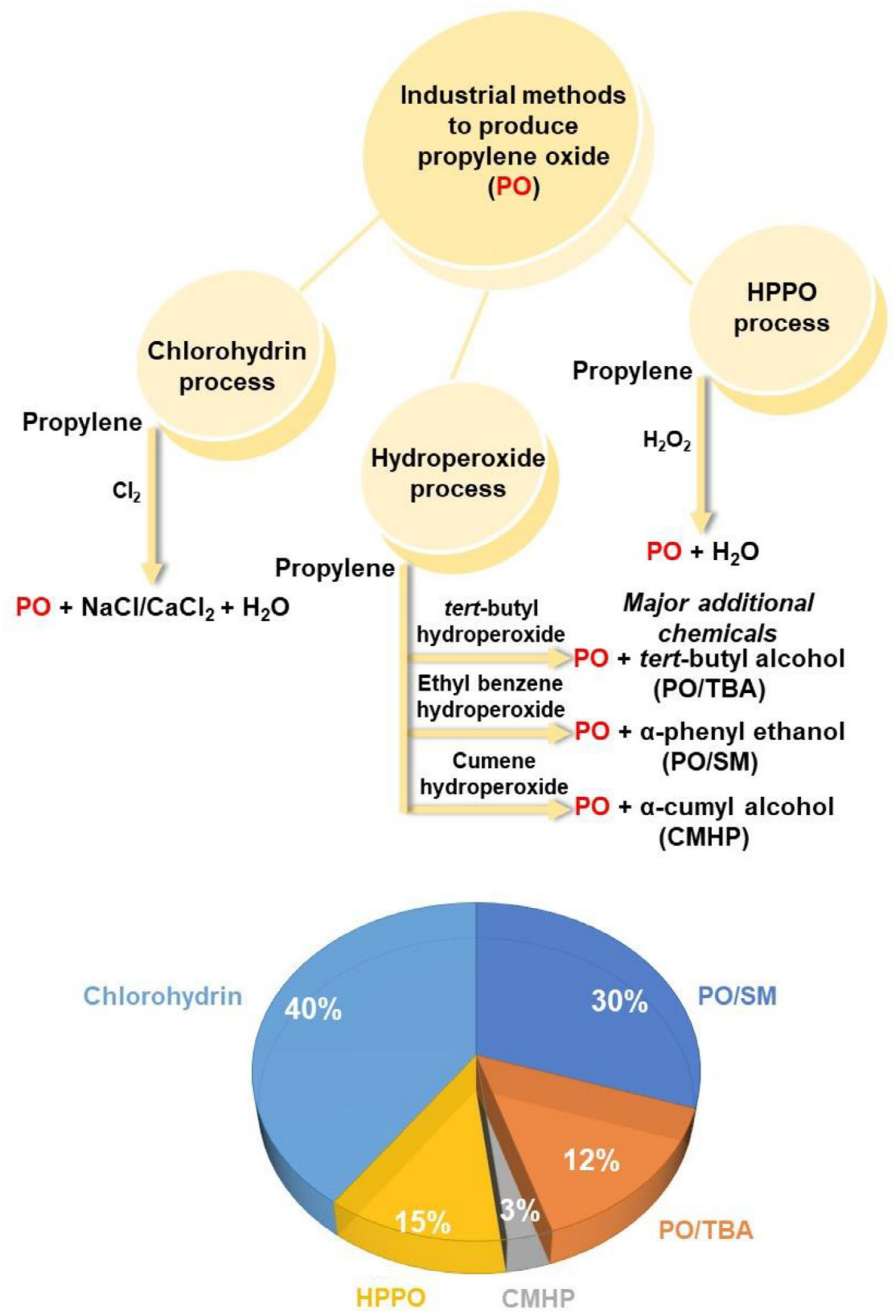
Industrial processes used for propylene oxide (PO) production and their respective percentage of the global PO production.

Various industrial processes have been developed to produce PO (Figure 1) and Table 2 provides a comparative summary of these methods,^[^ ^39^ ^]^ evaluating their industrial feasibility, environmental impact, technological maturity, and associated benefits and limitations, which will be discussed in the following sections.

**Table 2:** Comparison of PO synthetic routes based on industrial viability.

Route	Industrial Viability	Environmental Impact	Technological Maturity	Key Advantages	Key Limitations
Chlorohydrin Process	Commercially established	High (wastewater, chlorinated byproducts)	Mature (since pre‐1960s)	Established technology; compatible with integrated chlor‐alkali plants	Large water use; hazardous waste; handling of chlorine; declining favor in new plants
PO/SM process	Commercial	Moderate	Mature	Styrene is a valuable commodity	Styrene market dependency; 2.2–2.5 kg styrene/kg PO
PO/TBA process	Commercial	Moderate	Mature	Effective integration with fuel additives	Environmental concerns with MTBE; 2.5–3.5 kg MTBE/kg PO
CMHP process	Commercial (since 2003)	Low	Emerging commercially	Avoids market dependency; good for areas with water scarcity	Complex process; higher catalyst sensitivity
HPPO process	Commercial	Low	Mature	Cleaner, simpler process; lower energy and water use; no coproduct dependency	High H_2_O_2_ cost; catalyst deactivation; safety and separation issues
Hydroperoxidation (HOPO)	Under development (promising)	Low	Research stage	Green, one‐step route; uses H_2_ and O_2_; sustainable potential	Low productivity; catalyst stability; low H_2_ utilization

One of the oldest is the *chlorohydrin process* (CHPO), which accounts for approximately 40% of PO production and involves the reaction of propylene with hypochlorous acid to create chlorohydrin.^[^ ^2^, ^9^ ^]^ The hypochlorous acid is generated in situ by reacting chlorine with excess water. Subsequently, the chlorohydrin is combined with a hydroxide (NaOH or Ca(OH)_2_) in water, resulting in the formation of PO. However, this reaction also generates a substantial amount of NaCl or CaCl_2_ (∼40%), which holds little or no commercial value. Consequently, a significant volume of alkaline water (brine) is released as waste. Additionally, the separation of all hydrocarbons from the wastewater stream proves to be challenging, leading to considerable economic, and environmental difficulties.

The second most widely utilized method for PO production is the *hydroperoxide process*. This method involves the indirect oxidation of propylene using organic hydroperoxides, leading to the generation of PO along with alcohol as a coproduct.^[^ ^2^, ^9^ ^]^ This process is primarily conducted in three main steps: peroxide formation, epoxidation, and valorization of the co‐products. The selection of different hydroperoxides depends mainly on the market demand for the coproducts. The three primary oxidants utilized commercially are *tert‐*butyl hydroperoxide (PO/TBA process), ethylbenzene hydroperoxide (PO/SM, SMPO), and cumene hydroperoxide (CMHP process). In the PO/TBA process (Figure 1), isobutane is non‐catalytically oxidized using oxygen or air to produce *tert‐*butyl hydroperoxide (TBHP), which then undergoes a reaction with propylene in the presence of a homogeneous molybdenum catalyst.^[^ ^40^ ^]^ This results in the formation of PO and *tert‐*butyl alcohol (TBA). The TBA produced is further converted to methyl‐tert‐butyl ether (MTBE), a gasoline additive that is sold commercially. This method contributes approximately 15% to global PO production. The PO/SM (SMPO) method is commonly used in industries and accounts for 30% of global PO production. In this process (Figure 1), ethylbenzene undergoes oxidation to form ethylbenzene hydroperoxide. This hydroperoxide subsequently reacts with propylene, leading to the production of PO and α‐phenylethanol. The epoxidation step is commonly conducted using either a homogeneous molybdenum catalyst or a heterogeneous titanium/silica‐based catalyst.^[^ ^9^ ^]^ The co‐product, α‐phenylethanol, is then subjected to dehydration to yield styrene, which serves as a precursor for the synthesis of various polymers, including polystyrene, styrene‐butadiene rubber, acrylonitrile butadiene styrene, and styrene‐divinylbenzene.^[^ ^41^ ^]^ The third process (CMHP) represents a comparatively recent development by Sumitomo Chemical Company, Japan, and contributes to 3% of global PO production.^[^ ^41^ ^]^ In this method, cumene is oxidized in the presence of air to form cumene hydroperoxide (CMHP). The CMHP subsequently reacts with propylene, resulting in the production of PO and α‐cumyl alcohol. The epoxidation step employs a catalyst composed of mesoporous silicon dioxide with titanium, known as the Sumitomo Ti catalyst.^[^ ^5^, ^42^ ^]^ The co‐product obtained in the reaction is hydrogenated to recover the starting material cumene.

These three processes have the advantage of being selective toward PO, while generating minimal waste products compared to the chlorohydrin process. However, the PO/TBA and SMPO methods produce equal amounts of co‐products, which may affect their profitability if the demand for these products decreases. Additionally, these methods require multiple steps, including recycling and purification, which can be time‐consuming and costly.^[^ ^43^ ^]^

The *hydrogen peroxide to propylene oxide (HPPO) process* has emerged as another popular route for PO production, using *externally* sourced hydrogen peroxide as a direct oxidant for propylene. This process accounts for approximately 12% of global PO production. This method has gained significant attention in recent years and has been independently commercialized by DOW‐BASF, Evonik Degussa GmbH‐ThyssenKrupp AG and SINOPEC.^[^ ^5^, ^9^, ^44^, ^45^ ^]^ To ensure economic viability, most of the hydrogen peroxide (H_2_O_2_) used in this process is produced onsite since commercially available H_2_O_2_ is not cost‐effective for industrial applications. One of the prevalent approaches for generating the required hydroperoxide involves the anthrahydroquinone autoxidation process, which involves oxidation and reduction cycles of 2‐ethylanthrahydroquinone.

In the HPPO process, the externally produced H_2_O_2_ reacts with propylene in the presence of a TS‐1 zeolite catalyst at temperatures of 40–60 °C and pressures of 1–3 atm, using methanol as a solvent. H_2_O_2_ interacts with the tetrahedral Ti(IV) sites in TS‐1 to form a Ti–OOH (peroxo‐titanium) intermediate, which facilitates the electrophilic transfer of oxygen to the double bond of propylene, yielding PO. This reaction is exothermic, with an enthalpy change (ΔH) of approximately − 151 kJ/mol.^[^ ^46^ ^]^ The liquid methanol plays a crucial role in dispersing the heat generated during this exothermic reaction, thereby offering a thermal advantage over the hydroperoxidation (HOPO) process where H_2_O_2_ is produced in situ (details in Section 3). Following the reaction, methanol is separated from the mixture and recycled for use in the epoxidation reaction. This process exhibits high carbon and oxygen selectivity, where carbon selectivity indicates how efficiently propylene is converted into PO, with minimal generation of unwanted carbon‐based byproducts, and oxygen selectivity reflects the effective utilization of hydrogen peroxide for oxidizing propylene to PO. The BASF‐Dow HPPO process has reported a carbon selectivity >98% and oxygen selectivity >95% due to minimal formation of by‐products like acetone or CO_2_.

Thus, the HPPO process offers significant advantages over the chlorohydrin process, as it is a cleaner method and does not result in the production of substantial coproducts. However, it still faces certain limitations and challenges. One crucial aspect that requires attention is the development of a cost‐effective and economical method for producing H_2_O_2_ on‐site. This would eliminate the need for transportation and storage costs associated with commercially available H_2_O_2_. Additionally, the process involves high energy consumption during the separation and recovery of methanol, which is used as a solvent.^[^ ^5^ ^]^ Addressing this energy consumption issue is a significant challenge. As a result, ongoing research efforts are focused on improving the PO yield and reducing the overall energy consumption in the HPPO process.^[^ ^47^ ^]^ Researchers aim to enhance the process's sustainability and economic viability by finding more efficient ways to generate H_2_O_2_ and optimizing the separation and recycling of methanol.^[^ ^48^, ^49^ ^]^ These efforts aim to make the HPPO process even more attractive for industrial‐scale PO production in the future.

## 3 Hydroperoxidation of Propylene to Propylene Oxide (HOPO) by Gold Supported on Ti(IV)‐Containing Supports

The HOPOprocess using H_2_ and O_2_ has gained significant interest as a green, straightforward, and environmentally friendly method for PO synthesis. This one‐step process operates at moderate to high temperatures and atmospheric pressure, with water being the primary by‐product. Ever since the independent discoveries of Hutchings, who demonstrated that gold nanoparticles are catalytically active for the hydrochlorination of acetylene to vinyl chloride, and Haruta, who showed the exceptional activity of gold in the oxidation of carbon monoxide, there has been an increasing interest in exploring gold supported on Ti(IV), as a catalyst for hydroperoxidation of propylene.^[^ ^50^, ^51^ ^]^

**Figure 2:**
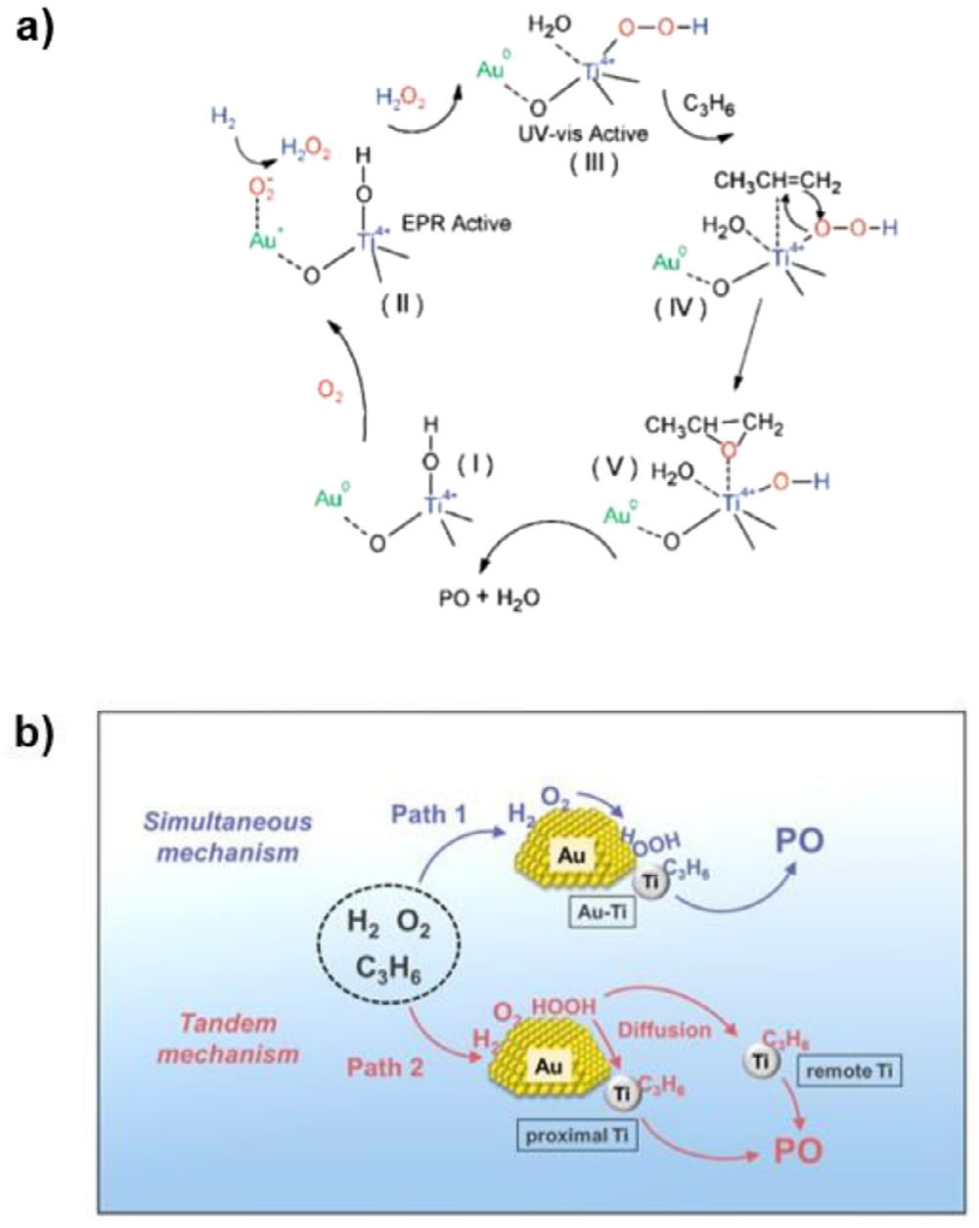
a) A possible mechanism proposed for the hydroperoxidation of propylene to PO. Reprinted with permission from ref. [53] Copyright 2008, American Chemical Society. b) A schematic showing simultaneous and tandem mechanisms for PO formation. Reprinted with permission from ref. [56] Copyright 2023, American Chemical Society.

The presence of both Au and Ti in close proximity to each other within the catalyst is crucial for the propylene epoxidation process to occur effectively.^[^ ^14^, ^52^ ^]^ Multiple spectroscopic techniques have been used by Bravo Suarez et al. to propose a possible reaction mechanism for propylene epoxidation with H_2_ and O_2_ on Au‐supported titanosilicates (Figure 2).^[^ ^53^, ^54^, ^55^ ^]^ This figure illustrates that when the Au is in close proximity with tetrahedral titanium (Ti) sites (species I), it facilitates the formation of H_2_O_2_ from hydrogen and oxygen over the Au surface (species II). The generated H_2_O_2_ is then transferred from the Au surface to nearby tetrahedral Ti sites, resulting in the formation of Ti‐hydroperoxo or peroxo species (Ti‐OOH) (species III). These Ti‐hydroperoxide species facilitate the epoxidation of propylene to PO (species IV). Subsequently, PO and water desorb from the catalytic sites (species V), regenerating species I. The common side products formed in this reaction are ethanal, propanal, acetone, acrolein, carbon dioxide, and water.

Additionally, Delgass and co‐workers employed DFT calculations to elucidate the mechanism involved in the propylene epoxidation using H_2_ and O_2_ over Au/TS‐1 catalyst.^[^ ^57^ ^]^ Their findings revealed that the formation of in situ hydrogen peroxide over small Au_3_ trimers, each with a diameter of approximately 0.3 nm, is energetically most favorable. These small Au nanoclusters can easily fit into the pores of TS‐1 (∼0.5 nm) and can also attach to the outer surface in close proximity to Ti sites, thus maximising the occurrence of the epoxidation step. By extension, this study suggests that small Au clusters are more active for propylene epoxidation. Zhou and co‐workers elucidated the role of Ti that is distant from Au for this reaction through a combination of experiments and kinetic modelling.^[^ ^56^, ^58^ ^]^ A kinetic model that incorporates both the tandem mechanism involving remote Ti sites and the simultaneous mechanism of proximal Au‐Ti exhibited the best agreement with experimental findings. They effectively demonstrated that remote Ti sites have the capability to capture active oxidant species diffused from isolated Au sites, thus playing a role in the epoxidation process (Figure 2).^[^ ^17^, ^34^ ^]^

These reaction mechanisms highlight the important role played by the catalyst in achieving high PO selectivity and yield. In a nutshell, this reaction involves two catalytic cycles occurring over two different active sites, Au and Ti, working in proximity: (1) in situ synthesis of H_2_O_2_ and (2) subsequent epoxidation of propylene by the generated H_2_O_2_. The latter step proceeds via Ti–OOH (peroxo‐titanium) species, similar to the mechanism observed in the HPPO process, where Ti–OOH intermediates enable electrophilic oxygen transfer to the double bond of propylene. Given the significance of both Au and Ti in this reaction pathway, a wide variety of Au‐Ti based materials have been developed for HOPO. Extensive efforts have been dedicated to deposit AuNPs onto Ti^4+^ based supports and ongoing research continues to explore and develop new and improved catalysts.

## 4 Efforts at *Nanoscale* and *Mesoscale*: Types of Support and Design of Supported Gold Catalysts

This section discusses key developments in catalyst design and support materials for the HOPO, focusing on both the nanoscale (e.g., gold nanoparticle size, preparation methodologies, promoters, and bimetallic nanoparticles) and the mesoscale (e.g., support types, porosity, and structural features), collectively. These two scales are closely connected, as the properties of the support can significantly influence the behavior and stability of the nanoparticles. The nature of the support not only provides a physical scaffold but also modulates the electronic and geometric properties of the active sites at the nanoscale. The synergistic interplay between these scales determines key performance metrics including activity, selectivity, hydrogen efficiency, and long‐term stability under reaction conditions.

### 4.1 Types of Supports Used in Propylene Epoxidation

**Figure 3:**
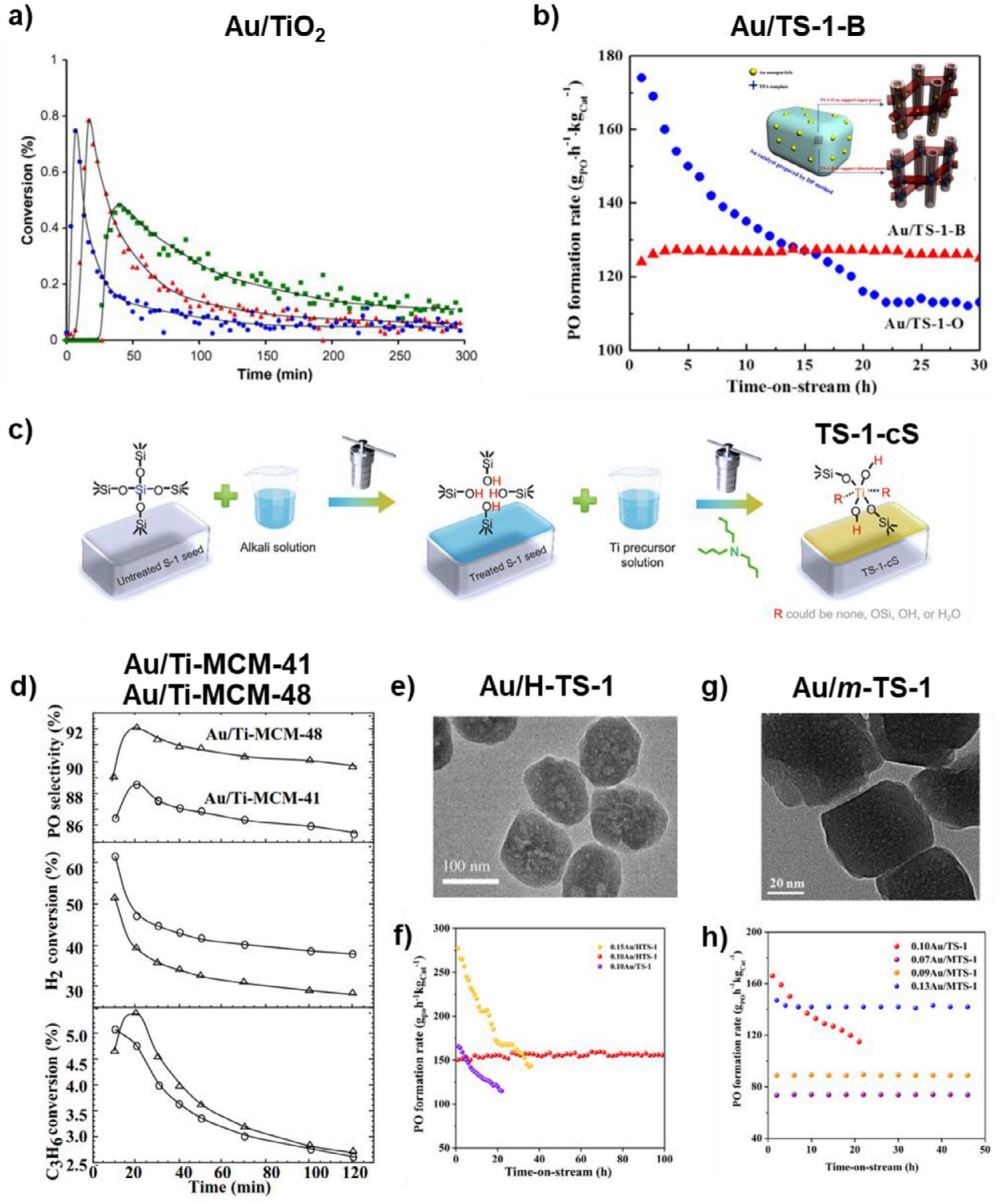
Mesoscale considerations: types of supports, and their influence on PO synthesis. a) Propylene gas phase hydro‐oxidation over 1 wt.% Au/TiO_2_ catalyst. Propylene conversion at 50 °C (green squares, PO selectivity ∼99%), 70 °C (red triangles, PO selectivity ∼96%), 90 °C (blue circles, PO selectivity ∼93%). Reprinted with the permission from ref. [6] Copyright 2006, Elsevier. b) PO formation rate of Au/TS‐1‐B and Au/TS‐1‐O catalysts as a function of the time‐on‐stream. Inset shows the schematic diagram of Au locations in Au/TS‐1‐O and Au/TS‐1‐B catalysts. Reprinted with the permission from ref. [67] Copyright 2014, Elsevier. c) Schematic diagram showing the synthesis of TS‐1‐cS catalyst, Reproduced with the permission from ref. [72] Copyright 2023, Elsevier; d) Catalytic performance of Au/Ti‐MCM‐41 and Au/Ti‐MCM‐48, Reprinted with the permission from ref. [73] Copyright 2002, Elsevier. e) TEM of H‐TS‐1 support, f) PO formation rate of Au/TS‐1 and Au/H‐TS‐1 catalyst at different time‐on‐stream. Reprinted with the permission from ref. [74] Copyright 2019, Elsevier. g) TEM of m‐TS‐1 support, h) PO formation rate of Au/TS‐1 and Au/m‐TS‐1 catalyst. Reprinted with the permission from ref. [75] Copyright 2017, American Chemical Society.

In 1998, Haruta and co‐workers reported their ground‐breaking discovery of the successful epoxidation of propylene using a catalyst consisting of AuNPs deposited on titania (P25 TiO_2_; combination of 78% anatase, 14% rutile, and 8% of an amorphous phase, specific surface area; 50 m^2^/g), with up to 1.1% propylene conversion and ∼99% PO selectivity at the reaction temperature of 50 °C.^[^ ^50^, ^52^ ^]^ Further spectroscopic studies revealed that the anatase phase of TiO_2_ plays an important role in the reaction and that Au/rutile TiO_2_ does not produce PO. Infrared spectroscopy studies by Nijhuis et al. proposed a reaction mechanism, which shows that Au facilitates the formation of bidentate peroxy species on Ti. Subsequently, hydrogen and oxygen react on AuNPs, leading to the formation of a hydroperoxide species.^[^ ^59^ ^]^ These peroxide species play a crucial role in facilitating the desorption of the bidentate propoxy species from the catalyst surface. Consequently, PO and water are generated, while the titania in the catalyst returns to its original state. Au/TiO_2_ catalyst suffered rapid deactivation within a few hours, due to strong adsorption of PO followed by the formation of PO oligomers on adjacent Ti sites through a bidentate propoxy intermediate, which accumulates on the catalyst surface (Figure 3a).^[^ ^6^ ^]^ The initially low activity of the Au/TiO_2_ catalyst illustrated in Figure 3a can be attributed to an induction period common in gold‐based systems,^[^ ^60^ ^]^ during which active sites and key species (e.g., H_2_O_2_ and Ti–OOH) gradually form, leading to low PO production. A comparison of the catalytic activities for different types of Au/Ti‐containing catalysts is shown in Table 3. Although this technology is still in the research and development phase and faces significant technical challenges that need to be resolved before pilot or commercial implementation, the key performance targets suggested for viability assessment are 10% propylene conversion, 90% selectivity to PO, and 50% H_2_ efficiency.^[^ ^61^, ^62^ ^]^ These will now be discussed in more detail.

**Table 3:** Summary of catalysts using different types of supports for propylene epoxidation.

Catalyst	Propylene conversion [%]	PO selectivity [%]	Temperature [°C]	H_2_ efficiency [%]	Au loading [wt.%]	Reference
Au/TiO_2_	1.1	99	50	‐	0.98	[52]
Au/TS‐1	1.1	99	150	5	1	[14]
Au/TS‐1	1	92	200	20	0.1	[43]
Au/TS‐1‐B	–	90	200	–	0.13	[67]
Au/U‐TS‐1	3.2	89.6	200	41.2	0.04	[68]
Au/TS‐2‐B	–	90	200	35	0.09	[76]
Au/Ti‐SiO_2_	0.08	99	60	–	0.39	[81]
Au/Ti‐SiO_2_	–	82.8	160	9.6	0.2	[83]
Au/Ti‐SiO_2_	–	93	130	39	0.08	[85]
Au/Ti‐MCM‐41	5.1	88	150	–	12	[73]
Au/Ti‐MCM‐48	5.6	92	150	–	12	[73]
Au/3‐D mesoporous titanisilicates	2.2	95.5	160	41	0.3	[61]
Au/H‐TS‐1		90	200	29	0.10	[74]
Au/Ti‐SBA‐15	0.04	100	50	12	1	[82]
Au/Ti‐TUD	0.9	99	150	17.4	0.07	[94]
Au/Ti‐MCM‐36	1.49	90.7	160	–	0.19	[89]
Au/Re‐ Ti‐MWW	2.6	81.9	150	–	0.16	[91]
Au/Ti‐YNU‐1	1.6	63.1	225	4.3	0.28	[90]
Au/m‐TS‐1		95.2	200	25	0.13	[75]
Au/TS‐1@*meso*‐SiO_2_	2.6	94.4	150	–	0.20	[100]
Au/TS‐1@S‐1	–	87.2	200	19.8	0.1	[103]
Au/S‐1/TS‐1@dendritic‐SiO_2_	4.18	93.9	200	26	0.1	[104]
Au/TS‐1‐M	6.1	91.7	160	13.6	5	[105]
Au/BTS‐1	14.1	88.1	200	45	0.48	[71]

Further research revealed significant enhancements in catalyst activity and stability when Ti was incorporated into a silica matrix. As a result, TS‐1 emerged as one of the most extensively studied supports.^[^ ^63^, ^64^ ^]^ In 1999, Nijhuis et al. reported successful dispersion of AuNPs on TS‐1, resulting in a suitable catalyst for propylene epoxidation with impressive PO selectivity exceeding 99% and 1.1% propylene conversion at 150 °C. The H_2_ efficiency for Au/TS‐1 was reported as 5% and the O_2_ efficiency (how effectively O_2_ is used in the formation of PO) as 7.9%. Notably, they observed that an increase in reaction temperature led to higher propylene conversion rates but a decrease in selectivity.^[^ ^14^ ^]^ In another approach, Delgass and co‐workers achieved a high PO formation rate of 160g_PO_ h^−1^ kg^−1^_cat_ at 200 °C using Au/TS‐1 (0.2 wt.% Au loading) catalyst by optimizing the pH and mixing time during catalyst synthesis.^[^ ^43^ ^]^ Furthermore, they also demonstrated that AuNPs located inside the nanopores of TS‐1 are dominant active sites for the PO reaction.^[^ ^17^, ^65^ ^]^ In another study, Delgass and colleagues conducted the first kinetic analysis of this reaction using a Au/TS‐1 catalyst.^[^ ^66^ ^]^ In order to obtain reliable kinetic data, the authors employed a design‐of‐experiments method that combined the efficiency of factorial experiments with the controlled variation of one‐at‐a‐time experimentation. A crucial aspect of this design was the explicit consideration of safety, with feed conditions carefully chosen to maintain operations outside the flammability region. They varied the oxygen concentration between 2 and 8 mol%, while hydrogen and propylene concentrations ranged from 8 to 24 mol%. The reaction was examined over a temperature range of 140 °C to 200 °C. The obtained kinetic data were empirically fitted to a power law rate equation:

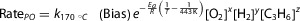




Here, 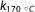 is the rate constant at 170 °C (the data were adjusted to the rate constant at 170 °C to reduce interactions between the preexponential factor and activation energy); Bias is a fitting parameter accounting for relative differences in the number of active sites; $E_{a}$ is the apparent activation energy; *R* is the gas constant; and $[O_{2}$]$,\;[H_{2}$] and $\left[C_{3}H_{6}\right]$ are the partial pressure of oxygen, hydrogen, and propylene, respectively. The exponents *x*, *y*, and *z* represent the apparent reaction orders for each reactant and were calculated as 0.31 ± 0.04, 60 ± 0.03, and 0.18 ± 0.04. Based on these fractional orders, they suggested that the rate‐determining step requires the participation of a minimum of two active sites, therefore, supporting a two‐site mechanism, where oxidant formation occurs on Au, while propylene reacts on Ti simultaneously. They explored the theoretical basis for a third site but found no statistically significant basis for it, and ultimately focused on a two‐site model for propylene epoxidation ensuring their experiments were carefully designed to mitigate flammability risks. Thus, the mechanistic rate expression for the PO production was as follows:

$$\begin{array}{ccc} \;\mathrm{Rat}e_{PO} & = & k_{6}\;zL_{2}{\left[K_{5}\left[C_{3}H_{6}\right]\right]}^{n}\left[\frac{K_{3}^{1/2}K_{4}{\left[H_{2}\right]}^{1/2}}{K_{1}^{1/2}K_{2}^{1/2}}\right]\\ & & \times \;{\left[K_{1}^{1/2}K_{2}^{1/2}K_{3}^{1/2}{\left[H_{2}\right]}^{1/2}\left[O_{2}\right]\right]}^{2m} \end{array}$$

Here, $k_{6}$ indicates the forward rate constant; z is the probability factor for finding adjacent surface intermediates; $L_{2}$ is the total number of active Ti sites; $K_{1},\;K_{2},\;K_{3},\;K_{4}$ are the equilibrium constants for elementary steps involving oxidant formation on Au sites; $K_{5}$ is the equilibrium adsorption constant for propylene on a Ti site; *n* and 2 *m* are the exponents resulting from the simplification of the Langmuir‐Hinshelwood‐type terms. The experimentally fitted reaction orders for propylene, hydrogen, and oxygen calculated above were found to be consistent with the simplified mechanistic rate expression when *n* = 0.18 and 2 *m* = 0.28. These findings highlight the critical role of a dual‐site mechanism that requires a minimum of two active sites (Au and Ti) functioning simultaneously in the rate‐determining step.

Feng et al. employed uncalcined TS‐1 (TS‐1‐B) where micropores of TS‐1 were blocked for propylene epoxidation.^[^ ^67^, ^68^ ^]^ The objective of blocking the micropores was to prevent any deactivation caused by PO adsorption within the micropores, and AuNPs were located on the external surface of the support.^[^ ^69^ ^]^ The synthesized Au/TS‐1‐B catalyst (0.12 wt.% Au loading) showed enhanced activity of 125 g_PO_ h^−1^ kg^−1^_cat_, ∼83% PO selectivity and high stability over 30 h as compared to Au/TS‐1‐O (open pores) as shown in Figure 3b. Furthermore, Wang et al. reported that a thermal treatment of Au/TS‐1‐B at 300 °C eliminates the extra TPA^+^ template from the external surfaces of the catalyst, which improves the PO selectivity and H_2_ efficiency concurrently.^[^ ^70^ ^]^ Recently, Sun and co‐workers employed a defect engineering technique to modify TS‐1 by selectively removing oxygen atoms adjacent to titanium atoms, creating a material termed as black TS‐1 (BTS‐1).^[^ ^71^ ^]^ This strategic elimination of oxygen atoms was designed to enhance the proximity between Au and Ti, facilitating the efficient transfer of *OOH species. As a result, a remarkable improvement was observed in both the PO production rate, which increased from 143 to 398 g_PO_ h^−1^ kg^−1^_cat_ and H_2_ efficiency, which rose from 26.6% to 45% compared to Au/TS‐1. Density Functional Theory (DFT) calculations further revealed that this modification significantly reduced the geometric distance between Au and Ti (from 3.0 Å to 2.6 Å) and drastically enhanced the electron‐accepting ability of Au (from 0.09 e to 0.21 e), contributing to improved catalytic performance.

**Figure 4:**
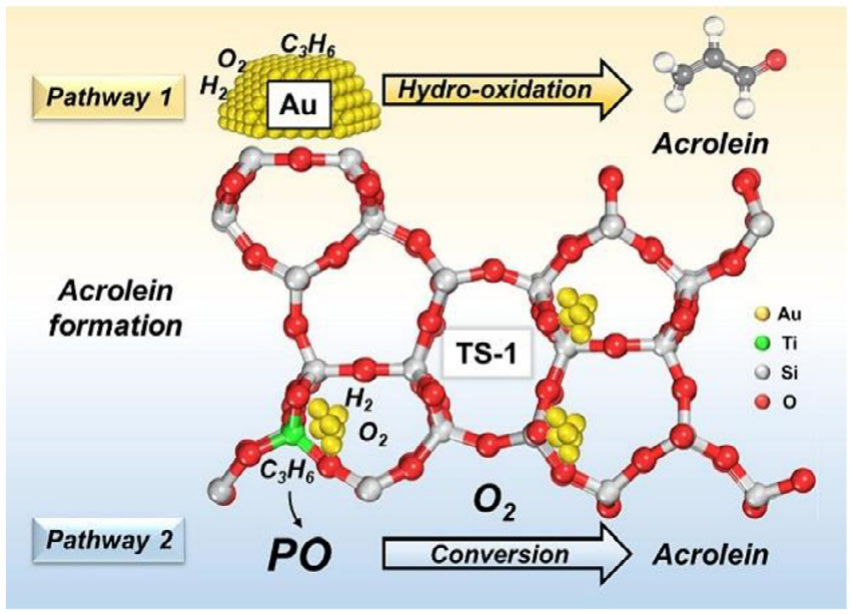
Schematic diagram representing reaction pathways proposed for Au/TS‐1 catalyst. Reprinted with the permission from ref. [80] Copyright 2024, Elsevier.

Besides TS‐1, Zhang et al. also reported the performance of uncalcined TS‐2 and compared it with uncalcined TS‐1.^[^ ^76^ ^]^ TS‐2 is known to possess defective Ti^3+^ sites along with Ti^4+^, which can facilitate the generation of hydroxy and superoxo species by activating H_2_O_2_.^[^ ^77^, ^78^ ^]^ The Au/TS‐2‐B catalyst, using a version of TS‐2 with blocked micropores, exhibited a PO formation rate of 118 g_PO_ h^−1^ kg^−1^_cat_ with 90% PO selectivity and high H_2_ efficiency of 35%.^[^ ^76^ ^]^ The PO formation rate over Au/TS‐2‐B catalyst was reported to be 1.6 times higher than that over Au/TS‐1‐B catalyst. The high catalytic activity was attributed to the unique synergy of Au‐Ti^4+^‐Ti^3+^ triple sites, where Ti^4+^ species are important in forming peroxo species resulting in more PO, while Ti^3+^ helps in the formation of peroxide radicals, which may eliminate the formation of carbonaceous species, therefore, enhancing the overall catalyst stability. Song et al. designed a strategy to create unsaturated Ti^3+^ sites on TS‐1 (TS‐1‐cS) by modifying silicalite‐1 (S‐1) seeds during hydrothermal synthesis. The S‐1 seeds are etched with varying concentrations of *n*‐butylamine, which increases the Si─OH content and grafts Bu_3_NH⁺ onto the seed surfaces.^[^ ^72^ ^]^ This treatment enhances nitrogen content and introduces structural defects, including Si─OH vacancies. During synthesis, these treated seeds are combined with *n*‐butylamine, TPAOH, colloidal silica, and titanium (IV) tetrabutoxide. Isomorphous substitution occurs between Si in the seeds and Ti from the precursor, resulting in TS‐1‐cS with coordinatively unsaturated Ti^3^⁺ sites. Bu_3_NH⁺ acts as an auxiliary template, promoting the formation of these Ti^3+^ species. The Ti^3+^ content in TS‐1‐cS increases with the Si─OH content in the seeds, indicating that these sites arise from substitution at defect locations. The schematic of this process is illustrated in Figure 3c. In another study, Zhou and co‐workers investigated the relationship between different crystal planes of uncalcined TS‐1 and propylene epoxidation. They concluded that with the decrease in TS‐1‐B crystal size there is an increase in PO formation rate, PO selectivity and as well as H_2_ efficiency, which was attributed to the enhanced Au‐Ti synergy.^[^ ^79^ ^]^ In a separate investigation, Du et al. conducted kinetic studies to elucidate the reaction pathways by comparing the catalytic performance of Au/TS‐1 and Au/TS‐1‐B.^[^ ^80^ ^]^ They examined the impact of two active sites present in the Au/TS‐1 catalyst, namely intrapore Au sites and external Au sites, in contrast to only one type of active site present in Au/TS‐1‐B, which comprises of external Au sites due to the blocking of micropores. Various feed ratios (total flow rate of 17.5, 35, and 50 mL minutes^−1^) and space velocities (7000, 14 000, and 20 000 mL g_cat_^−1^ h^−1^) were employed to study the catalytic performance. In both catalysts, the propylene conversion decreased with increasing gas hourly space velocity (GHSV), indicating that a higher residence time of PO on the catalyst bed could enhance PO conversion. Interestingly, it was observed that acrolein selectivity increased in TS‐1 with feed ratios of H_2_/O_2_/C_3_H_6_/N_2 _= 10/10/0.5/79.5% by volume, suggesting that acrolein forms as a primary product, which is not observed in TS‐1‐B when all were co‐fed at the same ratio, implying the role of micropores within the TS‐1 support (Figure 4).

In addition to TS‐1 and TS‐2, there has been considerable interest in using titanium‐modified mesoporous silicas (Ti‐SiO_2_) as support materials for propylene epoxidation. Furthermore, the incorporation of Ti externally into the silica framework can be achieved through either direct hydrothermal synthesis or a grafting procedure. This external incorporation of Ti facilitates quick desorption of PO.^[^ ^81^, ^82^ ^]^ The performance of the Au/Ti‐SiO_2_ catalyst was dependent on the calcination temperature of the support, and it was observed that with a higher calcination temperature of ca. 800 °C, there was an increase in the PO selectivity. A Au/Ti‐SiO_2_ (0.39 wt.% Au loading) catalyst exhibited 99% PO selectivity with 0.08% propylene conversion. Chen et al. have reported the effectiveness of Au/Ti‐SiO_2_, which exhibits stable PO reaction rates of approximately 120 g_PO_ h^−1^ kg^−1^_cat_.^[^ ^83^ ^]^ This catalyst was obtained by directly grafting Ti on SiO_2_, so that all the Ti sites were accessible to Au, which also suppressed the formation of propane. Following this work, Kanungo et al. achieved higher catalytic activity of 155 g_PO_ h^−1^ kg^−1^_cat_ at 220 °C with 88% selectivity to PO, by subjecting the Au/Ti‐SiO_2_ material to silylation, which helped to increase surface hydrophobicity, along with decreasing the number of Brønsted acid sites, and simultaneously increasing Au dispersion.^[^ ^84^ ^]^ To improve the performance of Ti‐SiO_2_ catalyst, Zhou and colleagues introduced an innovative method to anchor tetra‐coordinated Ti sites onto micropore‐free silica by cleaving surface hydroxyl and siloxane bridges at 1000 °C, thereby creating under‐coordinated oxygen sites that selectively stabilize active Ti⁴⁺ species.^[^ ^85^ ^]^ This approach increases the proportion of Ti^4+^ essential for the formation of Ti‐OOH intermediates in epoxidation. The resulting Ti‐SiO_2_ catalyst demonstrated enhanced hydrophobicity (minimising PO adsorption and promoting desorption), reduced acidity (limiting PO isomerization into by‐products), and strong synergy with Au sites, all contributing to superior catalytic performance.

Other popular mesoporous modified silica supports used in this study are Ti‐SBA‐15, Ti‐MCM‐41, Ti‐MCM‐48, Ti‐TUD, Ti‐MCM‐46, Ti‐MWW and Ti‐YNU‐1. Uphade and co‐workers demonstrated that Au/Ti‐MCM‐41 and Au/Ti‐MCM‐48 catalysts displayed high initial propylene conversion rates (5.1% and 5.6%, respectively) with corresponding PO selectivity (88% and 92%, respectively) at 150 °C.^[^ ^73^ ^]^ However, over a 2‐hour time‐on‐stream period, the conversion values declined significantly, reaching 2.6–2.7%, which is linked to the agglomeration of AuNPs and inaccessibility of active sites as shown in Figure 3d. To overcome this issue, Sinha et al. introduced 3D mesoporous titanosilicates with wormhole‐like mesoporosity, which offers several advantages over MCM‐41 including improved thermal stability, better diffusion of reactants and more efficient Au dispersion for increased accessibility of the active sites.^[^ ^61^, ^86^ ^]^ The catalyst exhibited high PO selectivity of 95.5% and H_2_ efficiency of 41%.

In another approach, Sheng et al. used hierarchical TS‐1 (H‐TS‐1) with wormhole‐like mesopores (ca. 45 nm) as a support and reported a high PO formation rate of 150 g_PO_ h^−1^ kg^−1^_cat_ and stability of 100 h as shown in Figure 3e,f.^[^ ^74^ ^]^ Yuan et al. demonstrated a sustainable route to synthesize H‐TS‐1 without additional mesoporogen.^[^ ^87^ ^]^ The Au/H‐TS‐1 catalyst exhibited a PO selectivity of 85% with PO production of 125 g_PO_ h^−1^ kg^−1^_cat_. In a further study, Sheng and coworkers optimized intracrystalline diffusion in Au/H‐TS‐1 using a two‐step recrystallisation approach.^[^ ^88^ ^]^ By employing mesoporogens of varying chain lengths (C8, C10, C12, and C16), they controlled the pore size of intracrystalline voids in TS‐1, with longer chains leading to larger voids. The void size increased from 4.1 nm to 19.5 nm with the C16 chain length. Additionally, catalyst stability improved significantly, extending from 6 h to 25 h due to enhanced intracrystalline diffusion when the longest chain length was used.

Among the various mesoporous modified silica supports, Ti‐SBA‐15 has been utilized as a catalyst support due to its superior hydrothermal stability, thicker walls, and larger pore size compared to MCM‐41 materials.^[^ ^82^ ^]^ Sacaliuc et al. reported 100% PO selectivity with 0.04% propylene conversion at 50 °C using Ti grafted Au/Ti‐SBA‐15.^[^ ^82^ ^]^ Higher propylene conversion of 1.1% was observed when the reaction temperature was increased to 150 °C, however PO selectivity decreased to 83.7%. These Ti‐SBA‐15 supported catalysts displayed considerable activity; however, they experienced rapid deactivation over time, typically within a few hours. Oyama and co‐workers demonstrated the catalytic activity of an Au/Ti‐TUD catalyst that gave a PO formation of 16.1 g_PO_ h^−1^kg^−1^_cat_ with 99% PO selectivity. Cheng and co‐workers used Au/Ti‐MCM‐36 with 2 nm mesopores as catalyst.^[^ ^89^ ^]^ A PO yield of 1.33% along with 91% PO selectivity was obtained using this catalyst. In another study, Cheng and co‐workers comparatively investigated Au/Ti‐YNU‐1 catalyst material with 0.67 nm micropores and obtained PO selectivity of 63.1% along with a PO production rate of 15.5 g_PO_ h^− 1^kg^−1^_cat_.^[^ ^90^ ^]^ They concluded that the stability of Au/Ti‐MCM‐36 catalyst was superior to that of Au/Ti‐YNU‐1, and this difference was attributed to the presence of 2 nm mesopores in Ti‐MCM‐36, as opposed to the 0.67 nm micropores in Ti‐YNU‐1, which facilitated the rapid diffusion of PO and prevented coke deactivation. Another mesoporous silica support that has been studied for this reaction is MWW. Ren *at al*. reported the application of MWW‐type of titanosilicate for the epoxidation and found that these Au/Ti incorporated MWW materials exhibited a PO formation rate of 9.1 g_PO_ h^−1^ kg^−1^_cat_ with low PO selectivity 57%.^[^ ^91^ ^]^ Further reducing the amount of Si─OH groups in the Ti‐MWW structure led to enhanced hydrophobicity, by which they were able to achieve a PO formation of 22 g_PO_ h^−1^ kg^−1^_cat_ and a PO selectivity of 81.9%.^[^ ^92^, ^93^ ^]^

The relatively low activity and faster deactivation in the Ti‐SiO_2_ support is mainly attributed to the adsorption of organic species on the surface of the catalyst that block the active sites and the aggregation of AuNPs, leading to decreased PO selectivity.^[^ ^94^ ^]^ To address the issue of diffusion limitations and subsequent adsorption of PO oligomers on the catalyst surface, Feng et al. have designed mesoporous titanium silicalite‐1 (*m*‐TS‐1) as support for this reaction with mesopores of 3 nm.^[^ ^75^ ^]^ The hierarchical structure of *m*‐TS‐1 improves the catalytic activity by inhibiting the extent of the side reactions on the catalyst, such as PO ring‐opening, which could otherwise lead to surface coverage and subsequent deactivation. Au/*m*‐TS‐1 catalysts exhibited high PO selectivity (>95%), high PO formation rate (142 g_PO_ h^−1^ kg^−1^_cat_) and good stability over 48 h (Figure 3g,h).^[^ ^75^, ^95^ ^]^

**Figure 5:**
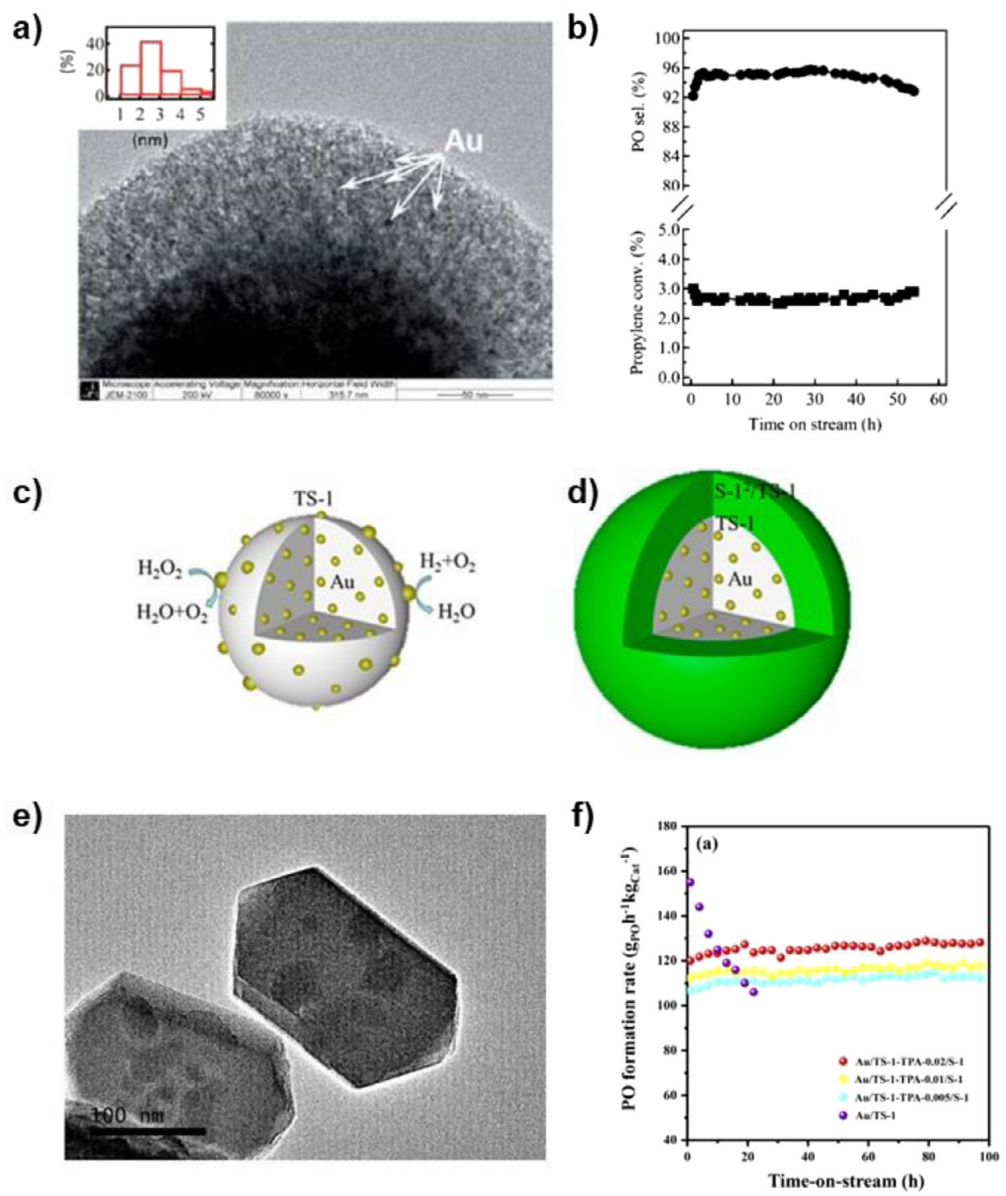
Various core‐shell supports used for propylene epoxidation: a) TEM of TS‐1@meso‐SiO_2_, b) propylene conversion and PO selectivity for TS‐1@meso‐SiO_2_, Reproduced with the permission from ref. [100] Copyright 2011, The Royal Society of Chemistry. Schematics showing dispersion of AuNPs in c) Au/TS‐1 and d) Au/TS‐1@S‐1. Reprinted with permission from ref. [102] Copyright 2019, American Chemical Society. e) TEM of TS‐1@S‐1. f) Comparison of PO formation rate for Au/TS‐1 and Au/TS‐1@S‐1 catalyst. Reprinted with the permission from ref. [103] Copyright 2019, Elsevier.

Core‐shell structures have also gained significant attention in recent years, as they offer several advantages, including enhanced stability, tunable properties and building synergy between various components.^[^ ^96^, ^97^, ^98^, ^99^ ^]^ These structures have also been explored as a catalyst for propylene epoxidation. Xu et al. developed core‐shell structured Au/TS‐1@*meso*‐SiO_2_ composite materials (Figure 5a), consisting of a TS‐1 zeolite core, a mesoporous silica shell, and Au nanoparticles (AuNPs) embedded in the mesochannels.^[^ ^100^ ^]^ These materials exhibited stable propylene conversion of 2.6% and PO selectivity of approximately 94% during a 54‐hour time‐on‐stream. Li et al. also discussed the improved stability of Au/TS‐1@S‐1 in comparison to traditional Au/TS‐1 catalysts for propylene epoxidation.^[^ ^101^, ^102^ ^]^ A schematic of this material is shown in Figure 5b. The significant advantage of these materials is that the confinement of Au particles into the mesopores of silica prevents their aggregation, thereby enhancing the overall catalyst stability. Yang and co‐workers synthesized a TS‐1@S‐1 support with Si rich cores and Ti shell by using minimal amount of TPAOH template.^[^ ^103^ ^]^ The Au/TS‐1@S‐1 catalyst showed a PO production rate of 126 g_PO_ h^−1^ kg^−1^_cat_ with 88% PO selectivity (Figure 5c). The enhanced catalytic activity is due to a hydrophobic core‐shell that reduces the mass transfer limitations and inhibits the surface blockage caused by PO oligomers. In another study, Song et al. developed a three‐layer core‐shell structure, denoted as S‐1/TS‐1@dendritic‐SiO_2_. The Au/S‐1/TS‐1@dendritic‐SiO_2_ catalyst exhibited exceptional PO selectivity of 93.9% and a stable PO formation rate of 143.4 g_PO_ h^−1^ kg^−1^_TS‐1_.^[^ ^104^ ^]^ The dendritic shell covers some Lewis and Brønsted acid sites, which appeared to enhance PO selectivity by inhibiting the PO ring‐opening reaction. Additionally, the middle layer of TS‐1 was identified to provide excellent mass transfer capabilities, thereby resulting in the catalyst's remarkable stability (>100 hours) during the reaction process. The transmission electron microscopy (TEM) images of different core‐shell structures used for epoxidation are illustrated in Figure 5.

Recently, Hou et al. prepared hollow microspheres of TS‐1 (TS‐1‐M) with mesopores ranging in size from 2 to 5 nm and 10 to 50 nm, derived from nano‐sized TS‐1 through the spray drying technique.^[^ ^105^ ^]^ Different silicon to titanium (Si/Ti) molar ratios of 30, 60, and 120, along with Au loadings of 1, 3, 5, and 7 wt.% were examined in this study. The hollow structure enabled rapid diffusion of the reactants and the products, leading to enhanced catalytic performance with a PO formation rate of 186.8 g_PO_ h^−1^ kg^−1^_cat_ and a PO selectivity of 92% achieved with Au loading of 5 wt.% and a Si/Ti molar ratio of 60.

### 4.2 Effects of Promoters and Pretreatments

**Figure 6:**
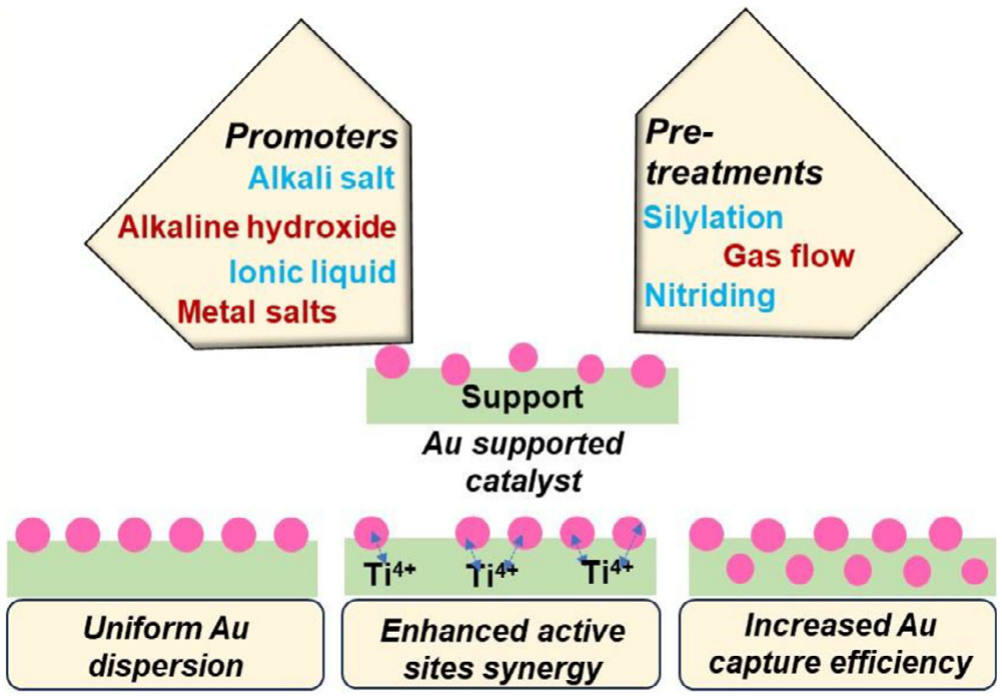
Schematic representation of how different precursors and pretreatments influence metal‐supported catalysts.

The addition of promoters and various pretreatments are used to enhance the performance of epoxidation catalysts by facilitating uniform dispersion of AuNPs, tuning the interplay between the active sites, and increasing Au capture efficiency (Figure 6).^[^ ^94^, ^106^ ^]^ Au capture efficiency refers to the support's capability to retain and stabilize Au on its surface, thereby preventing agglomeration or leaching, which can affect the final Au loading and overall catalyst performance. Table 4 presents the catalytic performance before and after the application of the promoter or pretreatment. In 2000, Haruta and coworkers demonstrated the use of CsCl as a promotor to enhance the catalytic activity of Au/Ti‐MCM‐41.^[^ ^107^ ^]^ The addition of CsCl via physical mixing to Au/Ti‐MCM‐41 catalyst improved the PO selectivity from 92% to 97%. It was hypothesized that CsCl supressed the reaction of H_2_ with O_2_. Subsequently, Sinha et al. reported superior performance of Au/3‐D mesoporous titanium silicate used Ba(NO_3_)_2_ as a promoter.^[^ ^61^ ^]^ The addition of this promoter encouraged the generation of hydroperoxide‐like species from H_2_ and O_2_, along with reducing the acidity of the catalyst. Qi et al. demonstrated that the catalytic activity and H_2_ efficiency of Au deposited on Ti‐doped nonporous silica were significantly enhanced through pretreatment in pure argon.^[^ ^108^ ^]^ The addition of other inorganic salts such as KNO_3_, LiNO_3_, sodium laurate (CH_3_(CH_2_)_10_COONa) and CsNO_3_ to the support is also known to enhance the catalyst performance.^[^ ^109^, ^110^ ^]^

**Table 4:** Catalyst performance before and after the addition of promoters or pretreatments. Values in red indicate performance after promoter (P) introduction.

			Propylene conversion (%)		PO selectivity (%)		H_2_ efficiency (%)		PO Production Rate		
Catalyst	Type of Promoter/ Treatment used (P)	Reaction Temperature (°C)	None	P	None	P	None	P	None	P	Reference
Au/Ti‐MCM‐41	**CsCl**	100	2.6	**1.4**	93	**97**	–	–	–	–	[107]
Au/Ti‐MCM‐41^[^ ^a^ ^]^	**Ba(NO_3_)_2_**	160	2.3	**6.3**	95.5	**92.3**	9.6	**34.6**	–	–	[61]
Au/TS‐1	**NH_4_NO_3_**	200	–	–	–	–	–	–	17	**104**	[111]
Au/TS‐1^[^ ^b^ ^]^	**(C_2_H_5_)_3_N**	150	4	**7**	88	**92**	15	**35**	–	–	[62]
Au/TS‐1	**Mg(NO_3_)_2_**	140	2.5	**3.9**	95	**90.9**	16.7	11.4	43	**65**	[112]
Au/TS‐1	**NaOH**	200	0.6	**7.4**	92	**85**	34	**30**	11	**119**	[113]
Au/TS‐1	**[BMIM][BF_4_]**	300	0.8	**10.1**	70.9	**72.1**	4.9	**2.76**	9.2	**120.2**	[115]
Au/TS‐1^[^ ^c^ ^]^	**NH_3_**	200	3.7	**7.4**	–	–	–	–	78.7	**138.4**	[116]
Au/TS‐1	**Na_2_CO_3_** **Cs_2_CO_3_**	200	0.6	**9.2**	65.3	**91.2**	5	**18.1**	27	**81**	[127]
Au/TS‐1	**Ni(NO_3_)_2_ ·6H_2_O**	170	3	**7**	88	**73**	42	**31**	85	**154**	[118]
Au/TS‐1	**NH_4_NO_3_/** **(NH_4_)_2_C_2_O_4_⋅H_2_O**	240	0.9	**11.9**	95.4	**84.5**	–	–	32.2	**366**	[119]
Au/TS‐1	**KHCO_3_**	180	3	**8**	60	**96.7**	10	**40**	30	**180**	[120]
Au/TS‐1	**Fe_2_O_3_**	200	–	**4** ^[^ ^d^ ^]^	91	**92**	–	**16** ^[^ ^d^ ^]^	90	**150**	[121]
Au/TS‐1‐B	**(NH_4_)_2_HPO_4_**	200	3.5	**6**	92	**89**	33^[^ ^e^ ^]^	**70** ^[^ ^e^ ^]^	112	**197**	[122]
Au/Ti‐SiO_2_	**Na₂SO₃**	200	0.8	**1.5**	60	**80**	10	**29**	38	**100**	[123]
Au/TS‐1	**NH_3_ + SBA** ^[^ ^f^ ^]^ **+ N_2_**	180	4.8	**6.8**	80	**96**	20	**40**	90	**160**	[124]

^[a]^ Silylated;

^[b]^ Silylated & Ba(NO_3_)_2_ treated;

^[c]^ nitriding of TS‐1;

^[d]^ reaction temperature of 180 °C;

^[e]^ Calculated at H_2_ conversion of 9%;

^[f]^ SBA‐ Silylation reagent triethoxyvinylsilane.

Cumaranatunge and Delgass demonstrated more than sixfold increase in PO production rate (104 g_PO_ h^−1^ kg^−1^_cat_) for Au/TS‐1 catalyst by using NH_4_NO_3_ when compared to untreated catalyst (17 g_PO_ h^−1^ kg^−1^_cat_).^[^ ^111^ ^]^ The Au‐amine complex formed during this treatment resulted in better Au capture, production of more in situ H_2_O_2_, hence, more formation of PO. Chowdury et al. showed that introducing small quantities of trimethylamine (TMA) as a cofeed further enhanced the hydrophobicity of the catalyst's surface.^[^ ^62^ ^]^ This, in turn, suppressed H_2_ combustion to H_2_O, effectively preventing the adsorption of organic species and resulting in improved catalytic stability. Lu et al. used Ba as a promoter to enhance the catalytic performance of Au/TUD catalyst.^[^ ^94^ ^]^ These alkalis facilitated the formation of small AuNPs (< 2 nm), which is crucial for this reaction.

Oyama and co‐workers reported the addition of alkaline earth metals, namely Mg, Ca, Sr, and Ba to boost the catalytic performance of Au/TS‐1.^[^ ^112^ ^]^ Best results were achieved when using Mg as the promoter, leading to a 51% rise in the PO formation rate compared to the untreated catalyst at a reaction temperature of 140 °C. The improved performance can be attributed to an overall increase in the Au capture efficiency by the additional charge possessed by the group 2 metal cations (M^2+^), which attracts the Au[(OH)_3_Cl]^−^ anions and promote better dispersion of AuNPs on the support material. In a separate report, Huang and colleagues utilized various alkali hydroxides, namely NaOH, LiOH, KOH, and CsOH, to pretreat TS‐1 before Au deposition. As a result, the formation of PO was remarkably increased by 11 times compared to the untreated catalyst.^[^ ^113^ ^]^ Subsequently, Lee *at al*. demonstrated that promotion by Cs led to better catalytic activity of Au/TS‐1 when compared to Na, K and Rb, due to the stronger interaction of Cs with AuNPs.^[^ ^114^ ^]^ Li et al. made a significant finding by employing the ionic liquid 1‐butyl‐3‐methylimidazolium tetrafluoroborate ([BMIM][BF_4_]) to improve the interaction between AuNPs and TS‐1.^[^ ^115^ ^]^ As a result, they achieved an impressive propylene conversion rate of 14.6% and a PO formation rate of 164.4 g_PO_ h^−1^ kg^−1^_cat_ using these materials.

**Figure 7:**
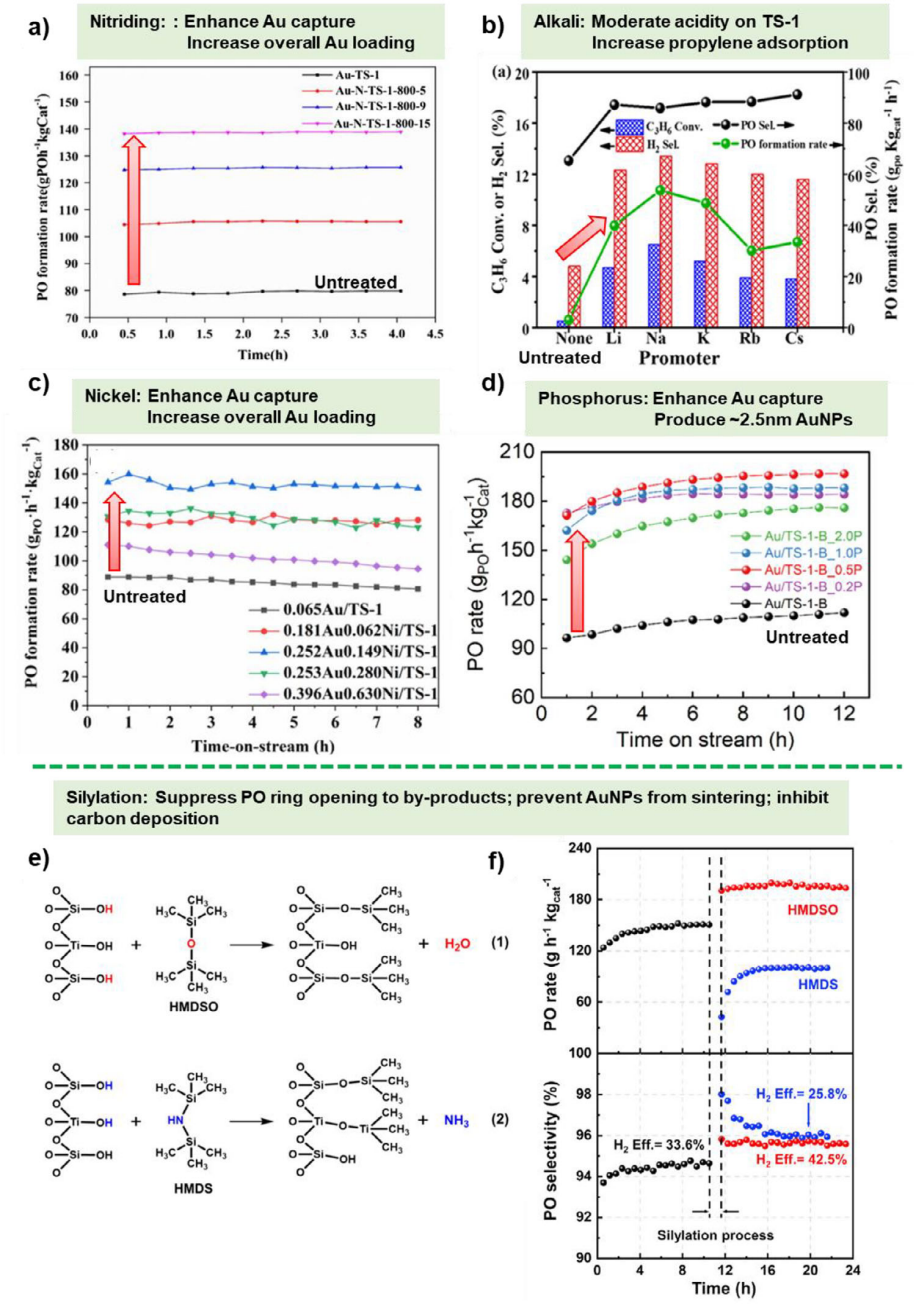
Effect of promoters and pretreatments on PO synthesis. a) PO Production rate of Au/TS‐1 catalyst before and after nitriding treatment at different time intervals; nitriding catalyst is denoted as Au‐N‐TS‐1‐x‐y where x is the nitriding temperature (°C) and y is the nitriding time (h). Reprinted with permission from ref. [116] Copyright 2020, Springer Nature; b) Catalytic performance of Au/TS‐1 catalyst using one or both promoting alkali metals, Na and Cs, during the preparation, Reprinted with permission from ref. [117] Copyright 2020, American Chemical Society, c) PO formation rate of untreated Au/TS‐1 and Ni treated Au/TS‐1 with different Au and Ni loading, Reprinted with permission from ref. [118] Copyright 2022, Springer Nature; d) PO production rate of Au/TS‐1‐B catalyst before and after P modification with varied P loading, Reprinted with permission from ref. [122] Copyright 2023, American Chemical Society, e) Equations representing the silylation of hydroxyl groups using HMDSO (1) and HMDS (2) and f) Catalytic performance after silylation using HMDSO and HMDS. Reproduced with the permission from ref. [125] Copyright 2021, Elsevier.

In another approach, Yuan et al. synthesized TS‐1 by incorporating nitrogen (N‐TS‐1), which is suspected to decrease the acid content on the catalyst surface and enhance its performance as shown in Figure 7a.^[^ ^116^ ^]^ The PO formation rate of Au/N‐TS‐1 catalyst (138.7 g_PO_ h^−1^ kg^−1^_cat_) was reported to increase by 77% as compared to Au/TS‐1 (78.7 g_PO_ h^−1^ kg^−1^_cat_). Yuegong and co‐workers discovered that incorporating two alkali metals, namely Na and Cs, significantly enhances the performance of Au/TS‐1 catalyst, surpassing the effectiveness of single alkali promoters (Figure 7b).^[^ ^117^ ^]^ With this catalyst, they achieved approximately 9.2% propylene conversion, coupled with an impressive 91.2% selectivity toward PO. Su et al. used Ni as a promoter for the Au/TS‐1 catalyst, resulting in improved Au capture efficiency and higher Au loading (Figure 7c).^[^ ^118^ ^]^ However, an excessive amount of Ni(II) led to the aggregation of AuNPs, and 0.154 wt.% was found to be the optimal value.

In a separate investigation, Dong et al. employed ammonium salt (NH_4_NO_3_ or (NH_4_)_2_C_2_O_4_) as a promoter to enhance the interaction of AuNPs with the support.^[^ ^119^ ^]^ A remarkably high PO formation rate was observed for NH_4_NO_3_‐promoted Au/TS‐1 (338.1 g_PO_ h^−1^ kg^−1^_cat_) and (NH_4_)_2_C_2_O_4_‐promoted Au/TS‐1 (285 g_PO_ h^−1^ kg^−1^_cat_). However, when both ammonium salts were combined for the pretreatment, the PO formation rate significantly increased to 366 g_PO_ h^−1^ kg^−1^_cat_, due to improved Au capture efficiency. Zhao and co‐workers used KHCO_3_ to improve Au capture efficiency during the synthesis of the Au/TS‐1 catalyst.^[^ ^120^ ^]^ This approach achieved 98.2% Au retention on the support, with only 1.8% loss. In contrast, the traditional DP method resulted in a significantly higher Au loss of 76.5% during the deposition step. KHCO_3_ improved the pH control that helped in the formation of desired [Au(OH)_x_]_3_ species instead of AuCl_4_^−^ in the Au precursor solution, which prevented agglomeration caused by Cl^−^ ions and enhanced the catalyst stability.

Ma and co‐workers developed a unique strategy to enhance the PO production by coating different metal oxides, namely CeO_2_, MnO_2_, Fe_2_O_3_ and Co_2_O_3_ onto the TS‐1 shell.^[^ ^121^ ^]^ They found that Au/TS‐1/CeO_2_ demonstrated a high PO formation rate of 160 g_PO_ h^−1^ kg^−1^_cat_ when the flow rate of O_2_ to H_2_ was 0.5 at 190 °C, attributed to the ability of CeO_2_ to adsorb a large amount of O_2_. It is noteworthy that some part of the adsorbed O_2_ is utilized for PO epoxidation, while the excess portion of O_2_ reacts with H_2_ to produce water. In a separate study, Xu et al. modified the TS‐1‐B support using ammonium phosphate followed by Au immobilisation to produce Au/TS‐1‐B_P catalyst.^[^ ^122^ ^]^ The phosphate treatment led to an enhancement in Au deposition from 69% to 88.9% and a reduction in the size of AuNPs from 2.9 to 2.4 nm, in comparison to untreated Au/TS‐1‐B. Moreover, phosphate facilitated the formation of highly active Ti sites and simultaneously enhancing the Lewis acid sites on the catalyst surface resulting in effective utilization of peroxo species to form PO. The PO formation rate for the phosphate‐treated catalyst increased by 76% as compared to the untreated catalyst, when the phosphorus loading was 0.002% as shown in Figure 7d. In addition to phosphorus modification, sulphation has emerged as a promising strategy for enhancing Ti‐SiO_2_ catalysts. Shi et al. employed an impregnation method using sodium sulphite solution to introduce sulphur species, leading to a favorable distribution of tetra‐ and penta‐coordinated Ti sites.^[^ ^123^ ^]^ The modified Ti‐SiO_2_‐S‐M sample with moderate sulphur loading showed an optimal Ti environment and increased Ti 2p binding energy, indicating lower electron density that enhances propylene adsorption. Sulphation also removed surface hydroxyl groups, improving hydrophobicity and promoting PO desorption while suppressing side reactions, as confirmed by DRIFTS. Additionally, it reduced both Brønsted and Lewis acid sites, decreasing PO isomerization and H_2_O_2_ decomposition, and increasing the availability of active Ti‐OOH species.

Among various treatment methods, Zhao et al. found that combining multiple pretreatments could effectively enhance catalytic stability.^[^ ^124^ ^]^ They reported that pretreating Au/TS‐1 with a mixture of 1% NH_3_, 1% triethoxyvinylsilane (silylation agent), and 98% N_2_ yielded significant improvements. Compared to a similar catalyst pretreated only with N_2_, the combined treatment showed notable improvements: propylene conversion at 100 h increased from 4.8% to 6.8%, PO selectivity improved from 80% to 96%, hydrogen efficiency doubled from 20% to 40%, and the PO production rate increased from 90 g_PO_ h^−1^ kg^−1^_cat_ to 160 g_PO_ h^−1^ kg^−1^_cat_. It is worth noting that the catalyst treated with the full combination remained stable up to 100 h, while the overall performance of the catalyst with only N_2_ treatment declined over time. The alkaline gas NH_3_ and silanisation treatments helped reduce acidic sites in Au/TS‐1, which were induced by TiO_x_ or extra‐framework Ti species. Additionally, triethoxyvinylsilane reduced the hydroxyl density on the TS‐1 surface, increasing its hydrophobicity, which facilitated easier desorption of PO. They also investigated the impact of different precipitating agents, including NaOH, K_2_CO_3_, and Na_2_CO_3_, and identified Cs_2_CO_3_ as the most effective, achieving the highest gold uptake efficiency.

The silylation technique has been shown to enhance catalytic performance by modifying surface properties. In this process, silanol groups react with alkyl silyl groups, such as methoxytrimethylsilane, increasing the material's hydrophobicity.^[^ ^84^ ^]^ This enhanced hydrophobicity facilitates the rapid desorption of polar products, thereby improving overall catalytic efficiency. Wang et al. used this approach to consume hydroxyl groups on the Au/TS‐1‐B catalyst using hexamethyldisiloxane (HMDSO) and hexamethyldisilazane (HMDS) as silane coupling agent.^[^ ^125^ ^]^ HMDSO was capable of consuming silanol groups without affecting titanol groups, while HMDS reacted with both silanol and titanol groups (Figure 7e). However, the reaction of HMDS with titanol groups led to a decrease in the Ti active sites and caused a reduction in catalytic activity. A long‐term assessment of the silylated Au/TS‐1‐B catalyst was conducted and HMDSO was introduced as a co‐feed into the reactor at a concentration of 50 ppm to uphold the hydrophobic properties of the catalyst's surface throughout the course of the reaction. The catalyst demonstrated a remarkable performance, yielding a PO formation rate of 193 g_PO_ h^−1^ kg^−1^_cat_, a PO selectivity of 95.7%, and an impressive H_2_ efficiency of 42.5%. The elevated catalytic activity was attributed to the chemical attachment of HMDSO, which selectively silylated silanol groups of the Au/TS‐1‐B catalyst (Figure 7f). This addition successfully restrained the agglomeration of gold particles as the reaction progressed, concurrently impeding the formation of coke owing to the inhibitory impact of silylation.

Among various catalyst treatment methods, thermal treatment is one of the most widely applied. The thermal conditions play a pivotal role in determining Ti configuration and, consequently, the catalytic performance. Conventional calcination in air at 550 °C often results in the transformation of active tetrahedral Ti^4+^ into inactive extra‐framework anatase TiO_2_ due to structural stress and localized overheating. This negatively impacts H_2_O_2_ utilization and increases the isomerization of PO into byproducts. Recently, Liao et al. demonstrated that tailored thermal treatments such as calcination under inert nitrogen atmospheres at lower temperatures can effectively suppress anatase formation, maintain active Ti sites, and enhance catalytic performance.^[^ ^126^ ^]^ These optimized TS‐1 samples exhibited lower extra‐framework TiO_2_ content (7–9% versus 10–14% in conventional TS‐1) and significantly improved PO formation rates (up to 121.63 g h^−1^ kg cat^−1^ compared to 98.49 g h^−1^ kg cat^−1^). Moreover, calcination under reduced oxygen concentrations further improved activity, underscoring the importance of controlling the oxidative environment. These enhancements were achieved without altering key structural properties like surface area, morphology, and MFI structure.

### 4.3 Various Catalyst Preparation Methodologies

**Figure 8:**
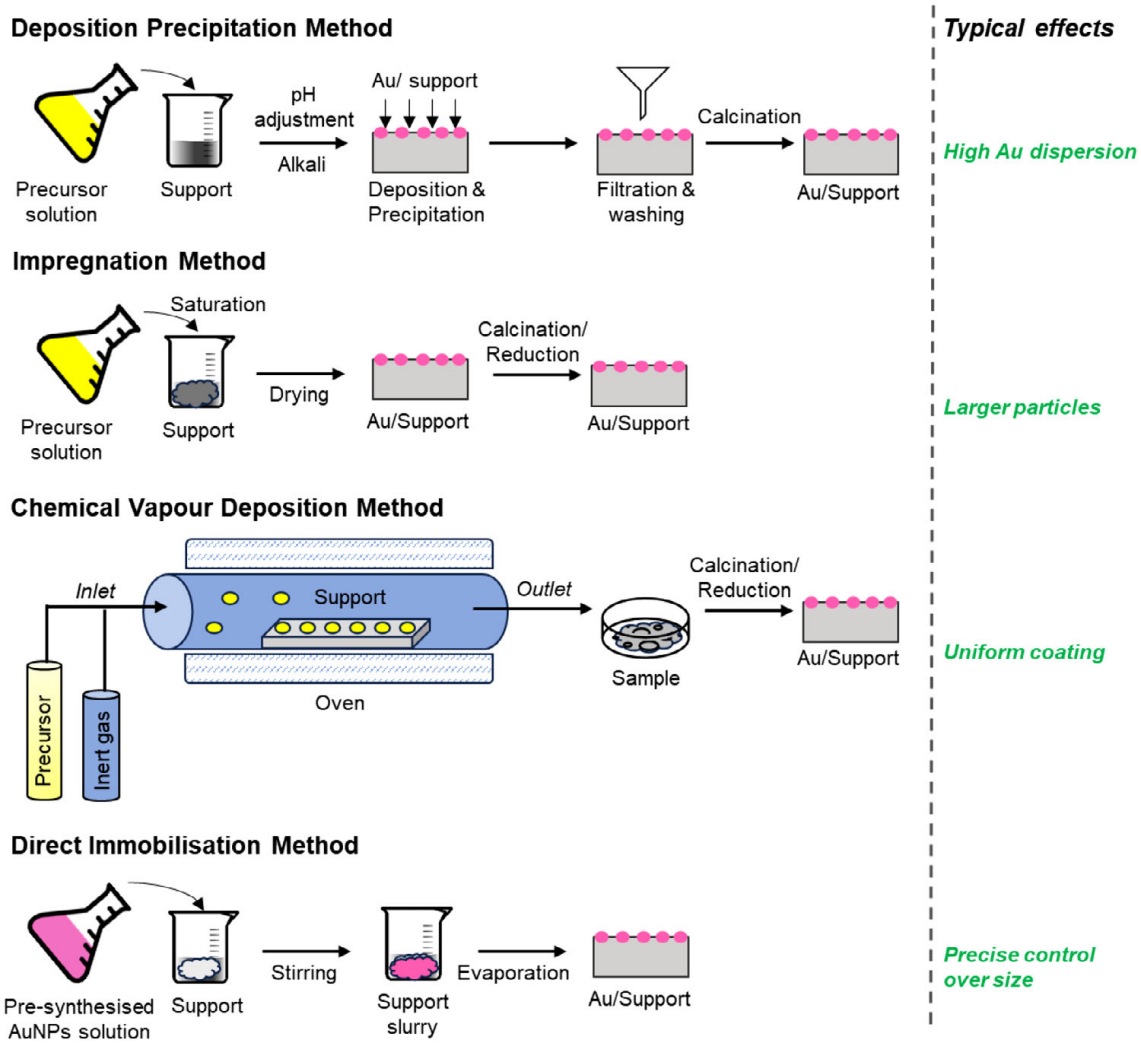
Schematic representation of various catalyst preparation methodologies for gold‐supported catalysts, including deposition precipitation (DP), impregnation, chemical vapour deposition (CVD), and direct immobilisation. Each method offers distinct advantages, such as improved metal dispersion, particle size control, and enhanced stability, influencing catalytic performance and application.

**Figure 9:**
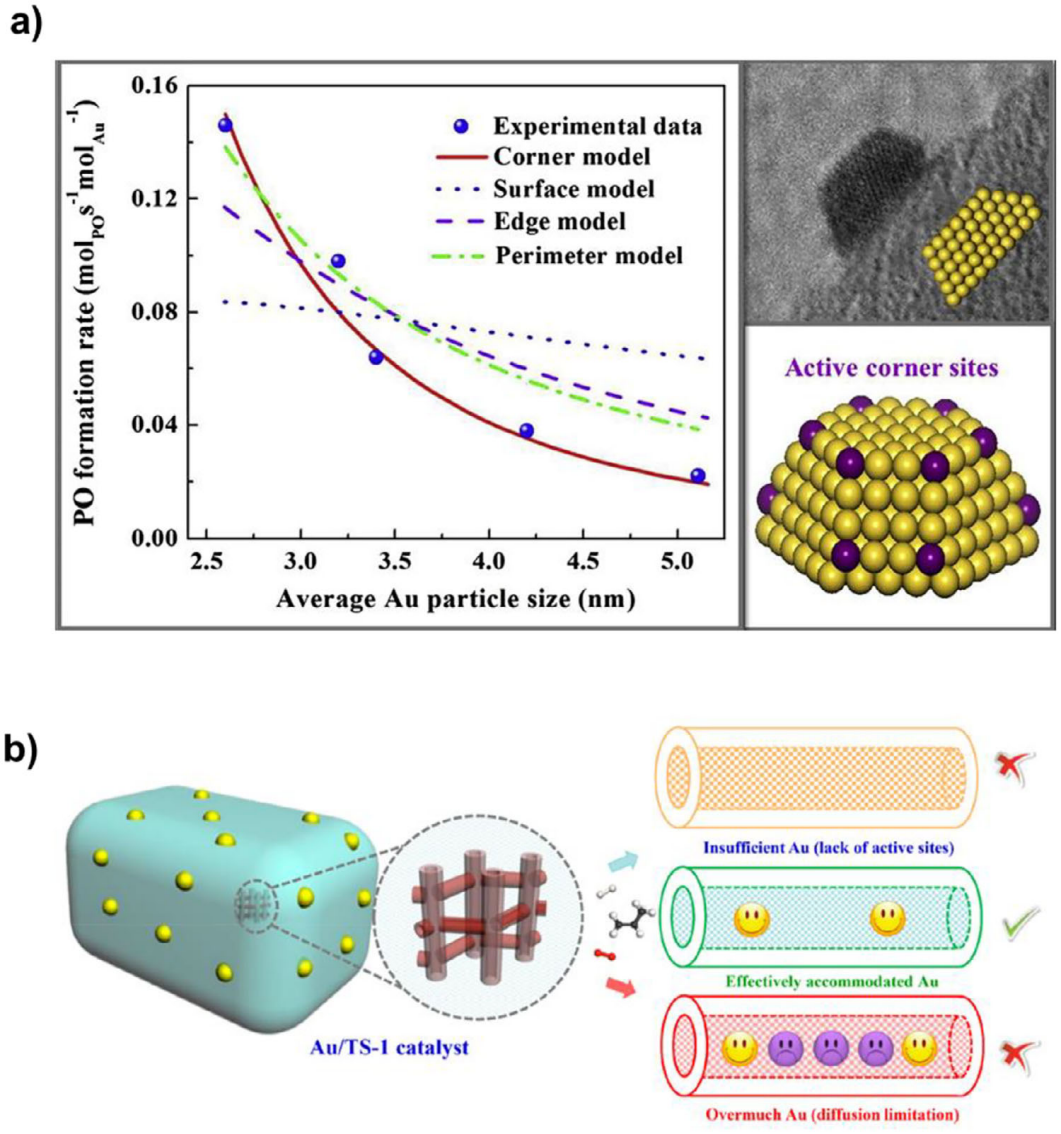
a) PO formation rate of uncalcined Au/TS‐1 catalysts derived from calculations and experimentations, along with a TEM image illustrating the typical truncated cuboctahedron shape of AuNPs. Reprinted with the permission of ref. [131] Copyright 2014, Elsevier. b) Schematic diagram of the position of AuNPs within TS‐1. Reprinted with permission from ref. [141] Copyright 2018, American Chemical Society.

The size, location, and geometry of AuNPs are crucial factors in propylene epoxidation, making the methods to prepare them very important.^[^ ^34^, ^128^, ^129^, ^130^ ^]^ A summary of different preparation methods used to synthesize Au/Ti containing catalysts is presented in Figure 8 and Table 5. A connection between the size of AuNPs and catalyst activity has been established indicating that a higher PO production rate was accomplished with smaller Au particles, primarily due to the increased fraction of corner sites that are dominant active sites for this reaction (Figure 9a).^[^ ^131^ ^]^ A different study affirmed that AuNPs with a small size of approximately 1 nm, whether situated within the nanopores of TS‐1 or on its external surfaces, hold a crucial function during the reaction.^[^ ^132^ ^]^ The most used method to prepare supported AuNPs is the deposition precipitation method (DP). This method involves adding a precipitating agent to the solution containing dissolved metal precursors (here HAuCl_4_), leading to the formation of nanoparticles through the process of precipitation. Haruta et al. conducted a comparison of distinct preparation methods, primarily focusing on deposition‐precipitation (DP), impregnation (IMP), and chemical vapor deposition (CVD), for the deposition of AuNPs onto various Ti‐based supports.^[^ ^50^ ^]^ Their findings led to the conclusion that the DP method is the most suitable technique for achieving Au/Ti‐based catalysts to selectively produce PO. In contrast, both IMP and CVD methods yielded larger‐sized nanoparticles, resulting in the complete oxidation of propylene and mainly generated CO_2_.

**Table 5:** Methods used to synthesize supported gold catalysts for propylene epoxidation.

Catalyst	Method	Particle size (nm)	Metal Loading (wt.%)	Precursor or ligand	Reference
Au/TiO_2_	DP	1–4	0.98	HAuCl_4_ (pH 7)	[52]
Au/TS‐1	DP	3–10	1	ammonia (pH 9–10)	[14]
Au/Ti‐SBA‐15	DP	6.5	1	ammonia (pH 9.5)	[82]
Au/Ti‐MCM‐41	DP	10–20	8	NaOH (pH ∼7)	[107]
Au/Ti‐SiO_2_	DP	2.4–3.5	8	NaOH (pH 7)	[81]
Au/TS‐1	DP	2–5	0.98–6.37	Na_2_CO_3_ (pH 4–10)	[139]
Au/TS‐1	DP	<1	0.1	Na_2_CO_3_ (pH 7.3)	[43]
Au/TS‐1	DP	<1	0.058	Na_2_CO_3_ (pH 6–8)	[132]
Au/TS‐1	DP	<1	0.314	Cs_2_CO_3_ (pH 6–8)	[132]
Au/Ti‐TUD	DPU	<2	≤0.05	urea	[134]
Au/TS‐1‐B	DPU	3.0 ± 0.5	0.1	urea	[70]
Au/TS‐2‐B	DPU	2.9 ± 0.4	0.09	urea	[76]
Au/ silylated‐Ti‐MCM‐48	LG	3.5 ± 0.4	0.5	dimethyl Au(III) acetylacetonate	[109]
Au/TS‐1	SG	<2	0.1	dimethyl Au(III) acetylacetonate	[113]
Au/TS‐1	DPU	5	1	urea	[143]
Au/Ti‐SiO_2_	DP	2.5–6^[^ ^a^ ^]^	1	ammonia (pH 9.5)	[83]
Au/TS‐1	BR‐ILEI	3.1–8.4^[^ ^b^ ^]^	0.5	*Cinnamomum camphora;* [BMIM][BF_4_]	[106]
Au/TS‐1	SEI	3.6	0.25	PVA	[149]
Au/TS‐1	DI	0.8	1	triphenylphosphine	[35]
Au/TiO_2_	DI	4.6	5	dodecylthiol	[151]
Au/TS‐1	mIWI	2.4	0.06	HAuCl_4_/NaOH (12)	[152]
Au/TS‐1‐B	IWI	2.8 ± 0.5	0.08	dithiosulfatoaurate	[156]
Au─Ti@MFI	SI	2.99 ± 0.4	0.5	*Cinnamomum camphora;* [BMIM][BF_4_]	[158]
Au/M‐TS‐1	one‐pot	2.6	1	3‐mercaptopropyl‐trimethoxysilane	[159]
an‐Au@TS‐1	SI	2.4	0.5	tannic acid/[BMIM][BF_4_]	[160]
Au/TS‐1‐B‐H_2_	SI	2.4 ± 0.5	0.1	polyvinyl pyrrolidone (PVP)	[161]

^[a]^ Size dependent on loading of titanium;

^[b]^ Size dependent on biomass concentration.

The DP method has been successfully used to deposit AuNPs onto various supports such as TiO_2_, TS‐1, TS‐1‐B, TiO_2_‐SiO_2_, Ti‐TUD, SBA‐15, Ti‐MWW, Ti‐YNU‐1, Ti‐MCM‐41.^[^ ^18^, ^91^, ^133^, ^134^, ^135^, ^136^, ^137^ ^]^ Among NaOH, NaHCO_3_, Na_2_CO_3_, NH_3_, and urea, NaOH was identified as the most effective precipitant for preparing Au catalysts supported on Ti‐MCM‐41, leading to the best yields of PO and H_2_ efficiency.^[^ ^138^ ^]^ The effect of gold precursor concentrations and pH variations was investigated while using Na_2_CO_3_ as a precipitating agent for TS‐1.^[^ ^139^ ^]^ It was observed that a pH range of 9–10 was most favorable for achieving maximum gold deposition with an average particle size of 2–5 nm. Besides Na_2_CO_3_, Cs_2_CO_3_ has also been employed as a precipitating agent.^[^ ^102^, ^140^ ^]^ The use of Cs_2_CO_3_ results in a three‐ to four‐fold enhancement in Au loadings, attributed to the improved Au‐Cs interaction.^[^ ^141^ ^]^ When Na_2_CO_3_ or Cs_2_CO_3_ were utilized in the preparation process, AuNPs with a size of less than 1 nm were successfully obtained.

In another study, Lee et al. examined the influence of the pH of the gold slurry solution, mixing time of gold solution and precipitating agent, and preparation temperature in the deposition precipitation (DP) process for Au/TS‐1. The best results were achieved with a pH of approximately 7.3 and a mixing time of 9.5 hours at room temperature.^[^ ^43^ ^]^ Urea is another effective precipitating agent utilized for depositing AuNPs, widely known as the DPU method. The advantage of using urea is its ability to prevent contaminants like Na^+^ on the catalyst surface.^[^ ^31^, ^142^ ^]^ Lu and co‐workers successfully used urea to deposit AuNPs onto TS‐1. High and uniform dispersion of AuNPs was achieved after the synthesis, which plays a key role in the epoxidation.^[^ ^143^ ^]^ In addition to TS‐1, the deposition of gold on TUD, TS‐1‐B, and TS‐2‐B has also been achieved using urea.^[^ ^70^, ^76^, ^79^, ^134^ ^]^ Ammonia has also been used for depositing gold on TS‐1, Ti‐SBA‐15, Ti‐SiO_2_ and TS‐1‐B.^[^ ^14^, ^83^, ^137^, ^144^ ^]^ Feng et al. conducted an in‐depth investigation into the precise positioning of AuNPs within the TS‐1 support using the DP method, achieved by adjusting the preparation temperature and ageing duration.^[^ ^141^ ^]^ They deduced that at lower temperatures (∼5 °C), a larger portion of Au complex tends to infiltrate the micropores of TS‐1. As a result, AuNPs on the external surfaces of TS‐1 were more uniformly distributed, substantially enhancing the catalyst's overall performance. Furthermore, it was also noted that an excess of AuNPs within TS‐1 pores could lead to pore diffusion resistance and subsequently diminish catalytic performance (Figure 9).

Lei and co‐workers utilized the atomic layer deposition (ALD) technique to prepare Au/TiO_2_‐SiO_2_ catalyst to precisely control the loading of TiO_2_.^[^ ^13^ ^]^ Through this approach, TiO_2_ was uniformly deposited onto SiO_2_ to create a TiO_2_‐SiO_2_ support. Subsequently, AuNPs were deposited via the DP method, employing NaOH as a precipitating agent. Wang et al. utilized a liquid grafting (LG) method to develop Au/silylated‐Ti‐MCM‐48, wherein Au(CH_3_)_2_(acac) was used as a precursor. The support was mixed with the precursor in acetone, refrigerated for 12 h, filtered, and then dried to obtain the final catalyst.^[^ ^109^ ^]^ The synthesized catalyst after calcination exhibited a PO selectivity of 88% and a PO yield of 0.98% at 250 °C. Biosynthetic AuNPs have also been used for the epoxidation reaction using bio‐reduction‐ionic liquid enhanced immobilisation (BR‐ILEI) method, where extracts from plant‐based materials like *Cinnamomum camphora* and *C. platyclade* were employed for the reduction of HAuCl_4_.^[^ ^106^, ^115^ ^]^ The TS‐1 support premixed with IL [BMIM][BF_4_] was added to the above solution to obtain well dispersed Au onto TS‐1.

Another method used to deposit small AuNPs is solid grinding, which involves mechanical grinding or milling solid gold precursors with a support material.^[^ ^37^, ^145^ ^]^ The resulting gold catalyst exhibited unique properties due to the controlled particle size and distribution achieved through the grinding process. Huang et al. employed dimethyl Au(III) acetylacetonate as the gold precursor to deposit gold onto TS‐1, leading to nearly 100% Au capture and the formation of very small gold particles with a size of less than 2 nm.^[^ ^113^, ^146^ ^]^ Sol‐enhanced immobilisation (SEI) is widely employed technique for the fabrication of gold‐supported catalysts, where AuNPs are prepared separately and subsequently deposited onto the support.^[^ ^21^, ^147^, ^148^ ^]^ Li et al. utilized this method by preparing polyvinyl alcohol (PVA) stabilize AuNPs in aqueous medium, and then mixed it with TS‐1 support, followed by filtration, rinsing thoroughly with water prior to their application.^[^ ^149^ ^]^ A comparative analysis of the performance of this SEI‐prepared catalyst was conducted in relation to counterparts created using the DP methods and direct impregnation (IMP). The catalyst, denoted as Au/TS‐1 (0.25 wt.% Au loading) and formulated through the SEI technique, showcased an elevated PO production rate of 20.17 g_PO_ h^−1^ kg^−1^_cat_, a notable improvement over IMP (0) and DP (2.88 g_PO_ h^−1^ kg^−1^_cat_). The reason behind this enhanced performance is attributed to the size of the AuNPs, as the SEI method yielded AuNPs with an average size of approximately 3.6 nm, in stark contrast to the 38.4 nm for IMP and 5.6 nm for DP. Moreover, the SEI technique also led to the attainment of a high and evenly distributed dispersion of AuNPs.

**Figure 10:**
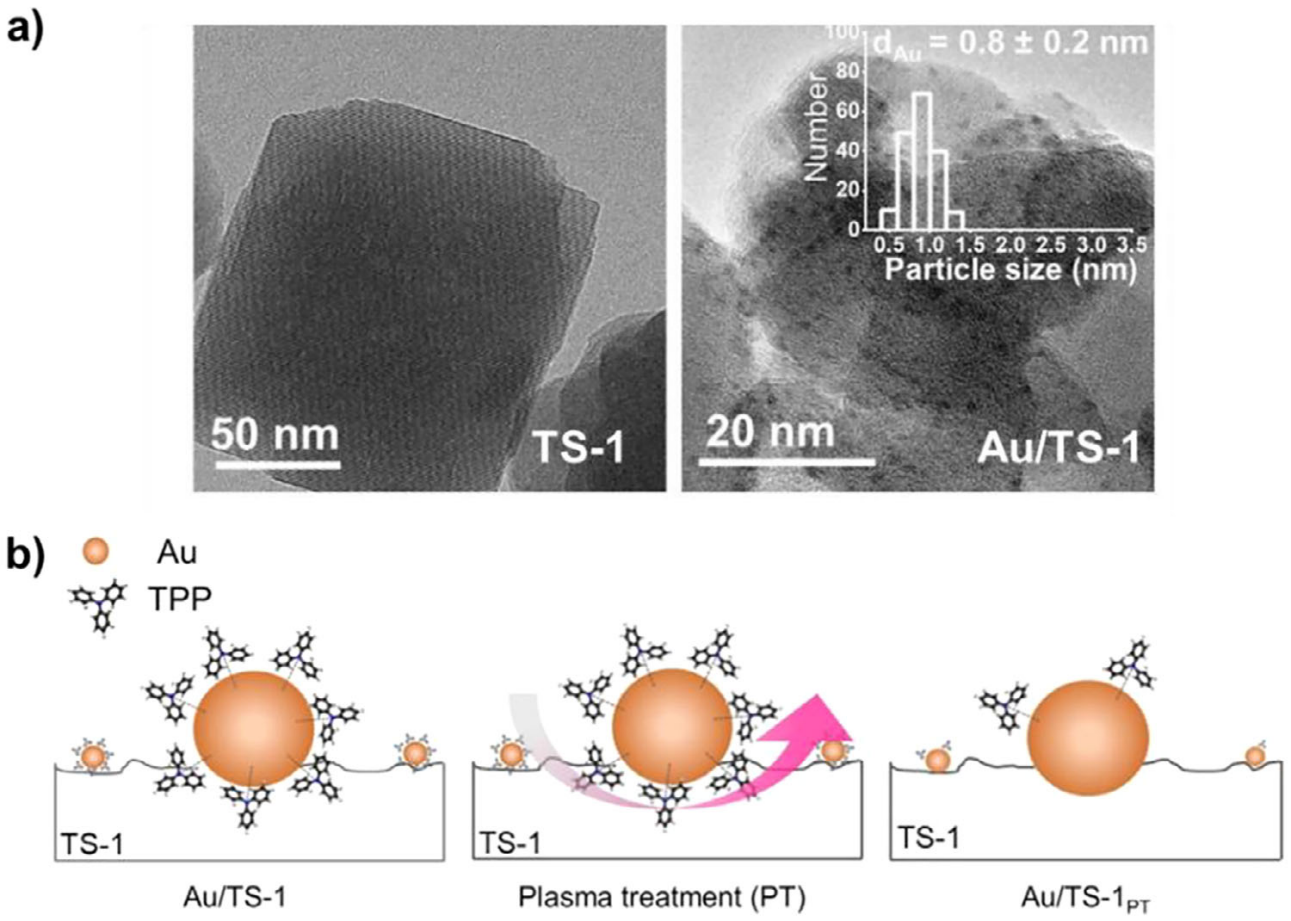
TEM image of a) TS‐1 and Au/TS‐1, with the gold nanoparticle size distribution shown in the inset, b) Schematic diagram showing the ligand removal effect of plasma treatment on Au/TS‐1. Reproduced with permission from ref. [35] Copyright 2021, Wiley‐VCH GmbH.

Kapil et al. introduced a straightforward one‐pot approach to fabricate atomically precise triphenylphosphine‐stabilized gold nanoparticles (AuNPs) of approximately 0.8 nm in size, which were then directly immobilized (DI) onto TS‐1 (Figure 10a).^[^ ^35^ ^]^ A versatile technique involving an O_2_ plasma was developed to partially remove the stabilizing ligands as shown in Figure 10b. The catalyst was regenerated by heating under 10% oxygen in helium at 300 °C for 1 h to remove any carbonaceous species that deposit on the catalyst's surface. The resulting catalyst exhibited a remarkable tenfold increase in stability for propylene epoxidation, in comparison to catalysts prepared using the DP method. This high stability, lasting around 20 days, coupled with a PO selectivity of 89%, was attributed to the small size of the AuNPs in close proximity to Ti^4+^ sites. Furthermore, the ligand played a pivotal role in preventing the aggregation of AuNPs. Additionally, they also investigated the impact of AuNPs size on propylene epoxidation activity.^[^ ^150^ ^]^ Different Au (I) precursors with varied steric hindrance around the phosphorus were employed to study the effect on final AuNP size. Notably, the AuNPs with the smallest size (∼2 nm) exhibited the highest PO formation rate and selectivity. Thiol‐capped AuNPs have also been directly immobilised onto TiO_2._^[^ ^151^ ^]^ The ligand was removed by calcination after the immobilization step. The synthesized Au/TiO_2_ catalyst exhibited 99% PO selectivity at a reaction temperature of 25 °C.

Lei and coworkers deposited Au onto TS‐1 using incipient wetness impregnation (IWI) with HAuCl_4_ (gold precursor).^[^ ^152^ ^]^ It has been well established that chloride ligands can trigger the aggregation of gold, resulting in suboptimal gold dispersion.^[^ ^153^, ^154^ ^]^ Therefore, the method was modified by incorporating alkali hydroxides (LiOH, NaOH, KOH, and CsOH) or alkali carbonates (Na_2_CO_3_, Cs_2_CO_3_) during the synthesis process. Among these, NaOH was identified as the most suitable choice, as it effectively eliminated any excessive chlorine content and facilitated the formation of small AuNPs. Na species are also known to prevent the formation of carbonaceous species onto the catalyst surface.^[^ ^155^ ^]^ Zhang et al. utilized a straightforward IWI approach in their work, employing dithiosulfatoaurate (Na_3_Au(S_2_O_3_)_2_) as the gold precursor to prepare the Au/TS‐1‐B (S‐Na) catalyst.^[^ ^156^ ^]^ Au/TS‐1 catalysts prepared using chorine‐containing gold precursors, specifically NaAuCl_4_ and HAuCl_4_, were also compared. The Au/TS‐1‐B (S‐Na) catalyst was found to exhibit a fivefold higher PO production rate compared to the reference catalyst. This enhanced catalytic performance is attributed to the strong interaction between Au and S atoms. Additionally, the absorbed sulphur species act as protective ligands, effectively preventing any agglomeration of AuNPs. In a latest study, they also examined the impact of thermal treatment at different temperatures (300 °C, 320 °C, and 360 °C for 2 hours) on the Au/TS‐1‐B (S‐Na) catalyst.^[^ ^157^ ^]^ The optimal temperature for thermal treatment was found to be 320 °C, which achieved a PO formation rate of 205 g_PO_ h^−1^ kg^−1^_cat_, along with high PO selectivity (93%) and hydrogen efficiency (36%). Treatment at 320 °C effectively facilitated the decomposition of S species adsorbed on the AuNP surface and the TPA⁺ templates on the external surface of TS‐1‐B. Higher temperatures led to an increase in AuNP size, while lower temperatures resulted in insufficient TPA⁺ decomposition. Guo et al. demonstrated that employing a freeze‐drying technique for drying the TS‐1‐B samples yields smaller and better dispersed AuNPs compared to conventional methods, such as oven‐drying and vacuum‐drying. This is attributed to the ability of freeze‐drying to prevent the aggregation of TS‐1‐B particles.^[^ ^154^ ^]^

**Figure 11:**
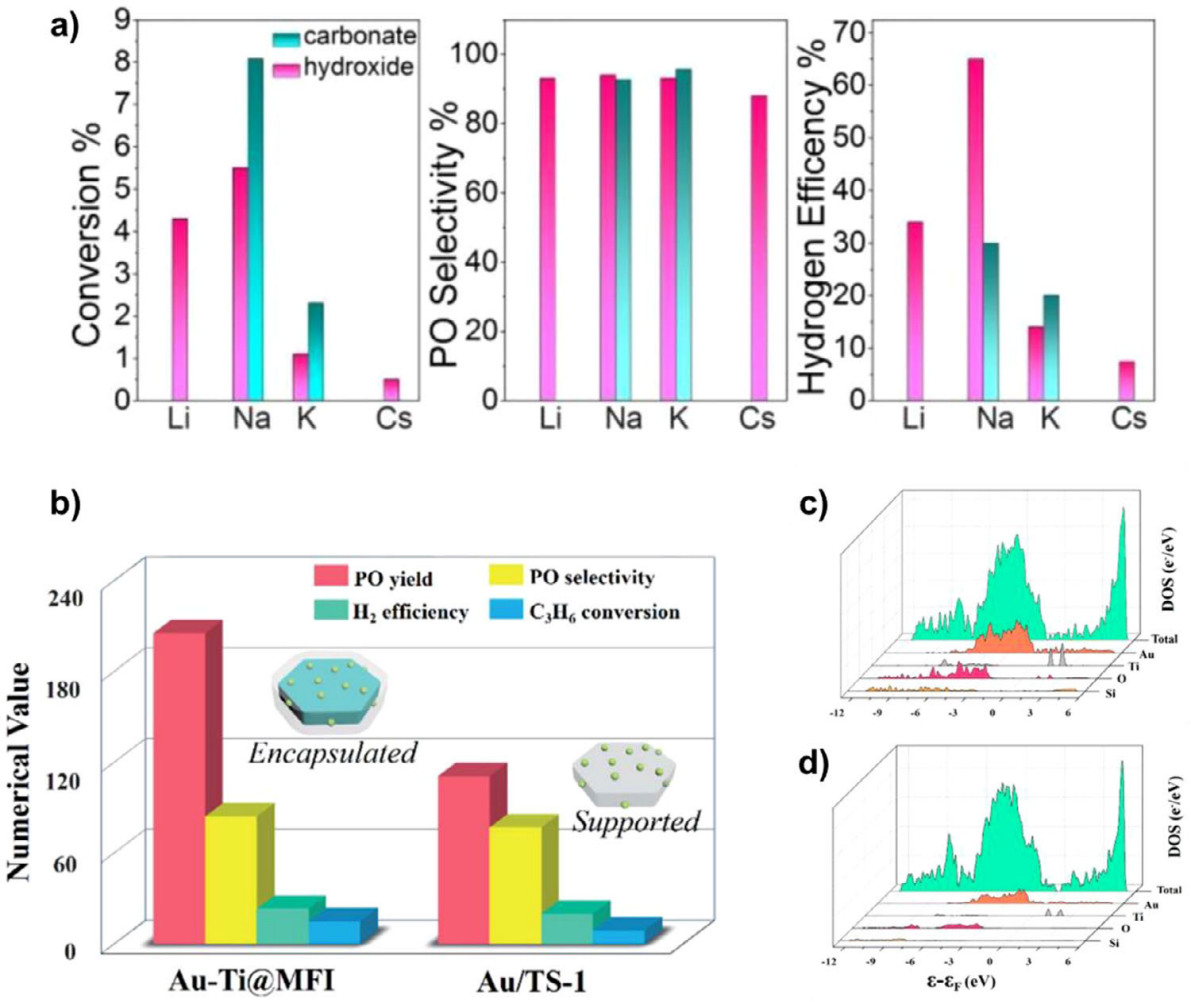
a) Catalytic performance, including propylene conversion, PO selectivity, H_2_ efficiency for Au/TS‐1 catalysts prepared using the various agents namely LiOH, NaOH, KOH, CsOH, Na_2_CO_3_, and Cs_2_CO_3_. Reprinted with permission from ref. [152] Copyright 2020, Wiley‐VCH GmbH. b) Catalytic performance including PO yield, PO selectivity, H_2_ efficiency, propylene conversion for Au─Ti@MFI and Au/TS‐1 catalysts, Density of states (DOS) of Au‐Ti interface in c) Au‐Ti@MFI and d) Au/TS‐1 catalyst. Reproduced with permission from ref. [159] Copyright 2020, The Royal Society of Chemistry.

The creation of enclosed AuNPs within a Ti‐containing support stands as a valuable technique for synthesising a stable gold‐supported catalyst. This encapsulation mechanism safeguards the nanoparticle size by preventing sintering at elevated temperatures. Wang et al. accomplished the synthesis of a zeolite‐encapsulated catalyst termed as Au‐Ti@MFI, wherein AuNPs were firmly anchored onto the Ti sites within titanium silicalite‐1.^[^ ^158^ ^]^ The preliminary step involved the preparation of Au/Ti/S‐1 through the sol‐immobilization method (SI). Subsequently, it was combined with SiO_2_ and treated with TPAOH. The resultant mixture underwent thermal treatment within an autoclave (180 °C for 72 hours), followed by calcination to yield the Au‐Ti@MFI catalyst. This catalyst showcased a PO formation rate of 205 g_PO_ h^−1^ kg^−1^_cat_ alongside a PO selectivity of 83.9%. Furthermore, DFT calculations were utilized to gain insights into the structural effects, revealing that the enhanced synergy between Au and Ti at the electronic level contributed to higher PO formation. When compared to Au/TS‐1, the density of states (DOS) of Au─Ti@MFI exhibited enhanced electron transfer between Au and Ti, primarily attributed to the increased overlap of Au─Ti states within the encapsulated catalyst (Figure 11).

**Figure 12:**
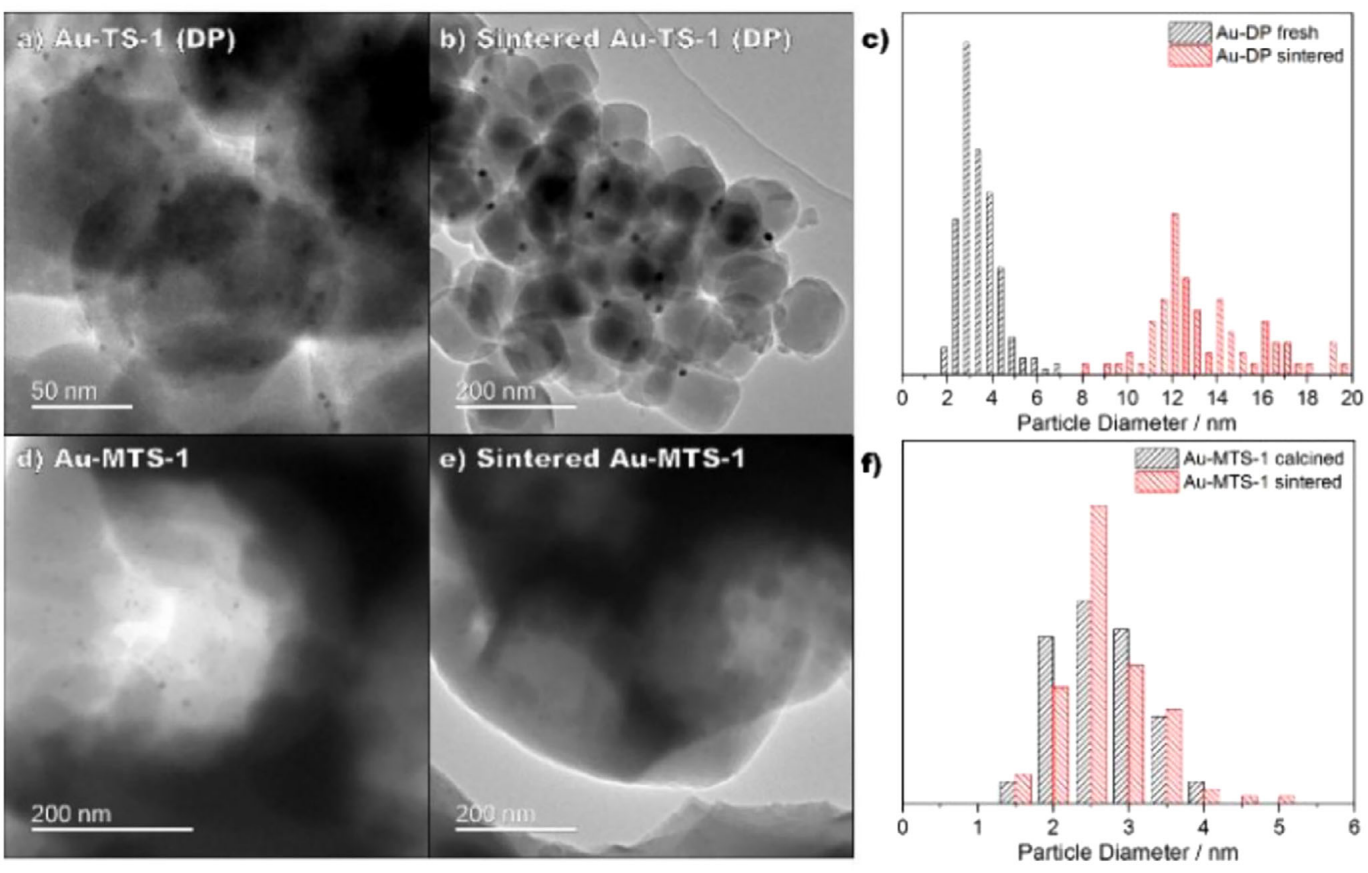
TEM images of a) Au/TS‐1 (DP), b) Au/TS‐1 (DP) after heat treatment c) Particle size distributions for catalysts shown in a & b. TEM images of d) Au/MTS‐1, e) Au/MTS‐1 after heat treatment, f) Particle size distribution for catalysts shown in d & e. The heat treatment was carried out at 800 °C for 18 hours. Reproduced with permission from ref. [160] Copyright 2022, Wiley‐VCH GmbH.

Weissenberger et al. reported a one‐pot procedure for fabricating hierarchical microporous zeolites that contained enclosed AuNPs (Au/MTS‐1). 3‐mercaptopropyl‐trimethoxysilane was used as a capping ligand to stabilize AuNPs.^[^ ^159^ ^]^ The catalyst was heated at 800 °C for 18 hours, and no alteration in the particle size was observed (Figure 12). The prepared catalyst was compared to Au/TS‐1 (DP), where clear agglomeration was seen after the heat treatment. This outcome indicated the remarkable stability of the AuNPs within the porous framework. Furthermore, the catalyst exhibited high activity but low selectivity toward PO. Wang et al. used tannic acid in the synthesis of Au/TS‐1.^[^ ^160^ ^]^ The phenol group within tannic acid played a crucial role in effectively integrating AuNPs with Ti within the catalyst's structure, a pivotal factor for selective PO formation. The resulting catalyst (an‐Au/TS‐1) demonstrated a remarkable 58% enhancement in the PO formation rate compared to the reference Au/TS‐1 catalyst.

Zheng et al. prepared Au/TS‐1‐B catalyst using an enhanced SI method, where Au was immobilized onto TS‐1‐B by reducing HAuCl_4_.3H_2_O using NaBH_4_.^[^ ^161^ ^]^ The AuNPs were stabilize using polyvinyl pyrrolidone (PVP). The primary advantage of this preparation method was achieving nearly 99.9% Au capture efficiency. Additionally, the catalyst was pretreated with H_2_ before the reaction to eliminate organic ligands, thereby enhancing the interaction between Au and Ti active site, which improved the catalyst performance and lifetime. Different types of preparation methodologies discussed in this section can help in producing AuNPs of different sizes. In this context, Arvay et al. performed an interesting study where they comparatively investigated the kinetics of propylene epoxidation of Au/TS‐1 prepared using different methods: Au‐PVP/TS‐1 and Au/TS‐1(DP).^[^ ^34^ ^]^ Despite differences in AuNP size and location (larger, extracrystalline particles in Au‐PVP/TS‐1 versus smaller, intraporous clusters in Au/TS‐1(DP), both catalysts show similar activation energies around 51 to 52 kJ mole^−1^ and comparable reaction orders for O_2_, H_2_, and propylene. This suggested that the active sites are energetically similar and follow the same reaction mechanism. However, the kinetics of hydrogen oxidation differed significantly, with Au‐PVP/TS‐1 showing a much lower activation energy and different oxygen dependence. Active site modelling suggested that distinct regions of the gold surface are involved in propylene epoxidation and hydrogen oxidation. These results imply a critical trade‐off: while smaller gold particles enhance PO selectivity by increasing interaction with Ti sites, they also facilitate unwanted hydrogen oxidation, which reduces overall H_2_ efficiency.

### 4.4 Application of Bimetallic Nanoparticles for Propylene Epoxidation

Bimetallic nanoparticles exhibit synergistic effects between the two metals, leading to improved catalytic activity compared to their individual components.^[^ ^162^, ^163^, ^164^ ^]^ Hence, the application of bimetallic nanoparticles has also been explored in the epoxidation of propylene. Table 6 illustrates the bimetallic nanoparticles that have been studied for this reaction. Moulijn and co‐workers attempted the addition of Pd and Pt to Au on TiO_2_/SiO_2_ support using the DP method. The Pd‐Au/TiO_2_‐SiO_2_ resulted in the formation of more propane, possibly due to monometallic PdNPs, while Pt‐Au/TiO_2_‐SiO_2_ showed a PO yield of 1.1% at a reaction temperature of 75 °C.^[^ ^165^ ^]^ Tsubaki and co‐workers used Au‐Pd bimetallic NPs by coating TS‐1 onto the surface of prepared Au‐Pd/TiO_2_@SiO_2_ catalyst resulting in a core‐shell structure. The resulting catalyst (TS‐1 zeolite coated Au─Pd/TiO_2_@SiO_2_) demonstrated propylene conversion of 10.61% and a PO yield of 0.31%, signifying the accessibility of Ti^4+^ sites.^[^ ^166^ ^]^ Corma and co‐workers employed Pd(Au)/S‐1 as a catalyst for direct PO synthesis, employing a one‐pot, two‐step approach.^[^ ^167^ ^]^ In the initial step, in situ H_2_O_2_ was generated using the catalyst, along with ammonium acetate as inhibitor and methanol as a cosolvent, which was succeeded by the introduction of propylene and supercritical CO_2_ into a stainless steel batch reactor. Subsequently, a mixture of H_2_ and O_2_ was added to the reaction, and the process was conducted for a duration of 5 h. The Pd_10_Au_10_/TS‐1 catalyst demonstrated a notable 75.5% PO selectivity and an 11.9% PO yield under reaction conditions of 60 °C temperature and 75 bar pressure. The solvent played a crucial role in this reaction.

**Table 6:** Catalytic performance of various bimetallic nanoparticle/Ti containing catalysts for the hydroperoxidation of propylene to PO.

Catalyst	PO yield (%)	PO production rate (g_PO_ h^−1^ kg^−1^_cat_)	PO Selectivity (%)	H_2_ efficiency (%)	Reaction temperature (°C)	Reference
Au‐Pd/TiO_2_‐SiO_2_/TS‐1	0.31	–	2.90	–	150	[166]
Pd_10_Au_10_/TS‐1	11.9	–	75.5	–	60	[167]
Au_10_Ag_1_/TS‐1‐B	–	174	84	44	200	[168]
Au_0.76_Pd_0.24_/TS‐1	–	40	45	–	200	[169]
Au_0.68_Pd_0.32_/TS‐1	–	1000^[^ ^a^ ^]^	50	3.5	200	[170]
Zr‐Au/TS‐1	–	350.3	91.7	∼10	200	[172]
Au_20_Pt_1_/TS‐1‐B	–	356	88	41	200	[173]

^[a]^ gold atom efficiency in g_PO_ h^−1^ g^−1^_Au_.

**Figure 13:**
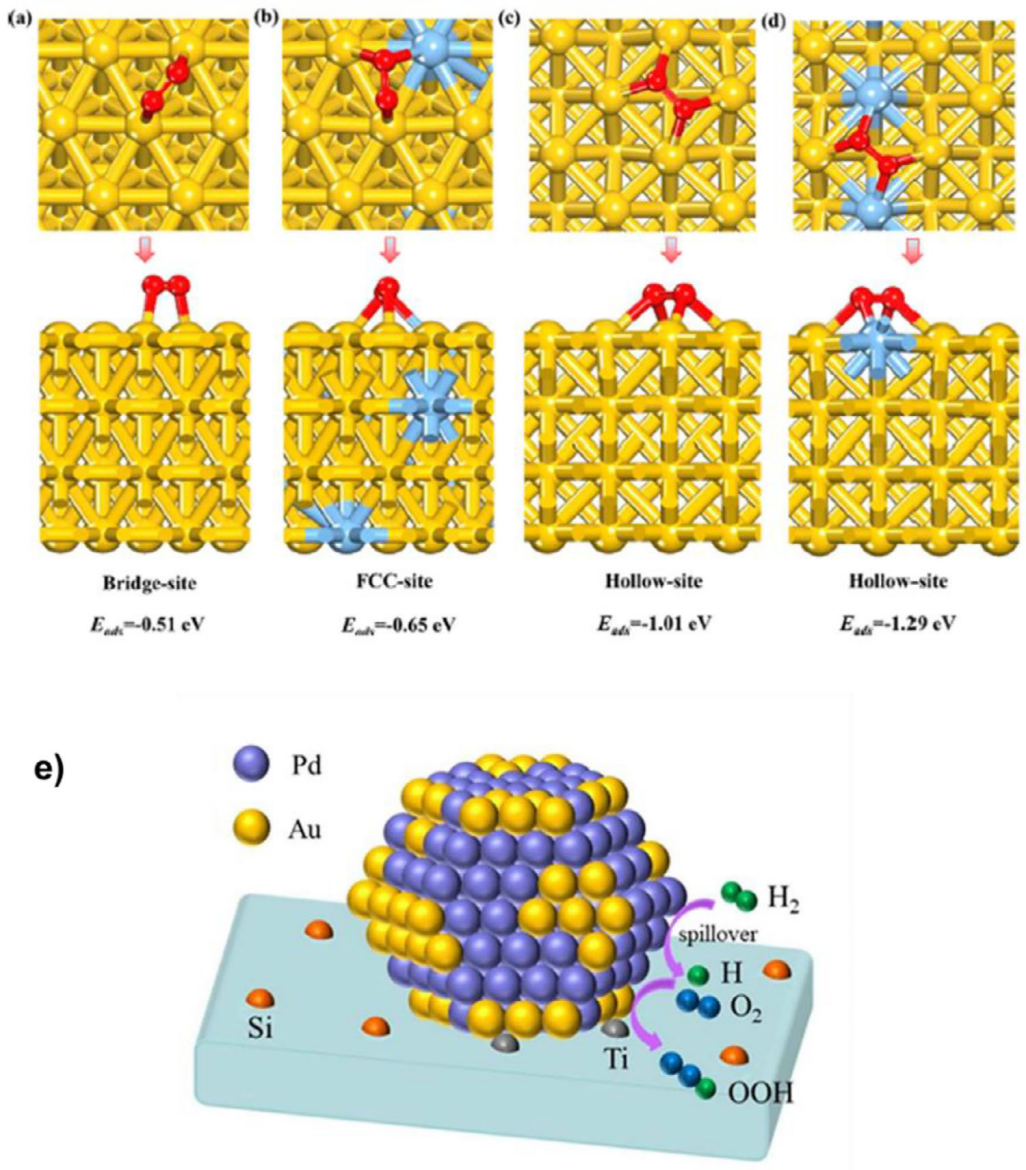
O_2_ adsorption configurations and adsorption energy for a) Au (111), b) Au − Ag (111), c) Au (100), and d) Au − Ag (100) calculated from DFT. Reprinted with permission ref. [168] Copyright 2018, American Chemical Society. e) Schematic diagram of H‐spillover phenomenon over Au‐Pd/TS‐1. Reprinted with permission from ref. [169] Copyright 2019, Wiley‐VCH GmbH.

Zhou and co‐workers reported that Au_10_Ag_1_/TS‐1 showcased superior catalytic activity in comparison to the Au/TS‐1‐B catalyst in the HOPO.^[^ ^168^ ^]^ This improvement was mainly due to the introduction of Ag, which served two important functions. First, Ag prevented Au‐Au agglomeration by forming well‐dispersed Au─Ag nanoparticles, reducing the average particle size from 3.2 nm to 2.7 nm and enhancing overall dispersion, which are crucial factors for achieving high catalytic activity. Second, Ag improved the oxygen adsorption and electron transfer properties of the catalyst. Furthermore, DFT calculations were employed to investigate the impact of Ag addition. The study involved the examination of O_2_ adsorption at four distinct sites (top, fcc, hcp, and bridge) on Au (111) surfaces. Notably, the most favorable adsorption site for O_2_ was found to be the bridge site with an adsorption energy (E_ads_) of 0.51 eV (Figure 13a). Subsequent calculations of O_2_ adsorption energies on Au (100), Au − Ag (111), and Au − Ag (100) surfaces at various sites revealed that O_2_ adsorption is stronger on the surfaces of bimetallic Au − Ag nanoparticles when compared to pure Au nanoparticles (Figure 13). These findings suggest that Ag promotes electron transfer from Au to O_2_ forming O_2_^−^ species that are key intermediates for the in situ formation of H_2_O_2_. This, in turn, enhances the overall rate of PO formation.

Li et al. investigated the effectiveness of Au‐Pd/TS‐1(Au loading 0.006 wt.%; Pd loading 0.001 wt.%) and noted a substantial enhancement in performance (with a PO production rate of around 40 g_PO_ h^−1^ kg^−1^_cat_) when compared to the pure Au/TS‐1 catalyst (with a PO production rate of approximately 1 g_PO_ h^−1^ kg^−1^_cat_; Au loading 0.007 wt.%).^[^ ^169^ ^]^ The increase in the PO formation is due to the phenomenon of H‐spillover from Pd atoms to Au atoms, which then reacts with O_2_ and, ultimately, the formed peroxo species in close proximity to Ti sites results in the epoxide production (Figure 13e). The ratio of Au and Pd also influenced the performance of the catalyst. In another investigation, they designed cuboctahedron‐structured Au‐Pd NPs, which facilitates effective charge transfer from Pd to Au atoms and subsequently to adsorbed O_2_ to eventually form in situ H_2_O_2_ by reacting with H_2_.^[^ ^170^ ^]^ The Au_0.68_Pd_0.32_/TS‐1 showcased a high gold atom efficiency of 1000 g_PO_ h^−1^ g^−1^_Au_ when compared to Au/TS‐1‐DP (105 g_PO_ h^−1^ g^−1^_Au_). Furthermore, an investigation into the reaction mechanism of the Au‐Pd/TS‐1 catalyst was conducted, unveiling a preference for propylene hydrogenation at Pd sites and propylene epoxidation at Au‐Ti sites.^[^ ^171^ ^]^ The introduction of 0.5% CO was found to suppress the propylene hydrogenation on Au‐Pd/TS‐1.

Peng et al. utilized Zr‐Au binary clusters immobilized onto the TS‐1 support as a catalyst. It was discovered that Zr functions as a stabilizing agent for Au, resulting in a particle size of approximately 0.6 nm.^[^ ^172^ ^]^ The catalyst demonstrated remarkable stability over 51 hours, achieving a propylene conversion rate of 10.56%, a PO selectivity of 91.42%, and a productivity of 350.30 g_PO_ h^−1^ kg^−1^_cat_. The Independent Gradient Model based on Hirshfeld partition (IGMH) technique was employed to assess the interaction stability between Au and Zr, revealing a robust interaction exceeding the van der Waals interaction between two Au atoms in the Au_2_ cluster and Zr. The presence of Zr prevents the migration and aggregation of small Au clusters into larger clusters. In another study, Wang et al. demonstrated that combining platinum addition with surface silylation significantly enhances the catalytic performance of Au/TS‐1‐B for propylene epoxidation.^[^ ^173^ ^]^ Introducing trace Pt into AuNPs (Au_20_Pt_1_/TS‐1‐B) increases the electron density at Au and Ti sites, promoting H_2_ dissociation and O_2_ activation. This facilitates the formation of hydroperoxide species by lowering energy barriers for OOH* and H_2_O_2_ intermediates, overcoming a key rate‐limiting step. Furthermore, the electron‐donating silyl groups enhance the electron density on Pt sites, decreasing propylene adsorption and hydrogenation. Together, these modifications create a favorable electronic and surface environment where Pt accelerates hydroperoxide formation while silylation promotes PO desorption and suppresses side reactions.

## 5 Epoxidation Using Nickel: An Alternative Catalyst to Gold?

**Figure 14:**
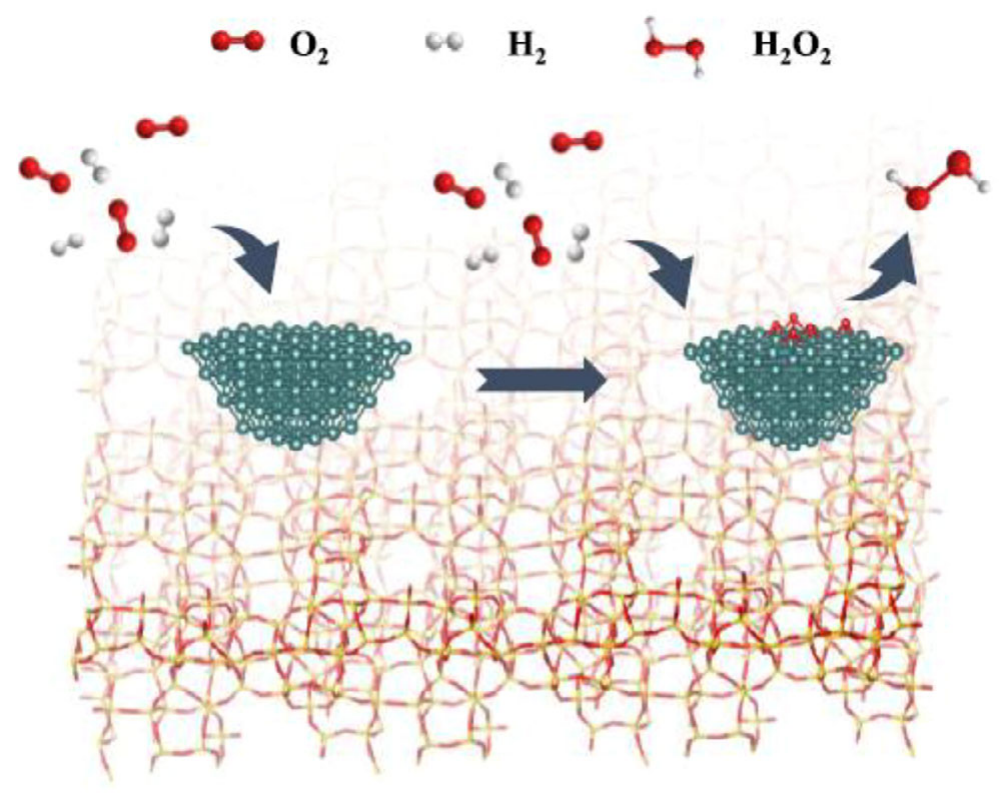
Illustration of H_2_O_2_ formation on Ni(111) surface. Reprinted with permission from ref. [175] Copyright 2023, American Chemical Society.

In recent years, there has been growing interest in identifying cost‐effective and sustainable alternatives to gold‐based catalysts for selective epoxidation reactions. Among these, nickel has emerged as a promising candidate due to its unique electronic and structural properties. García‐Aguilar and co‐workers introduced a novel approach by employing NiNPs instead of AuNPs for HOPO.^[^ ^174^ ^]^ The Ni/Ti‐SiO_2_ catalyst (0.5 wt.% Ni loading, particle size ∼3 nm) demonstrated a C_3_H_6_ conversion of 6.3%, PO selectivity of 85.5%, and H_2_ efficiency of 37%, similar to the performance of an Au/TS‐1 catalyst (8.8% C_3_H_6_ conversion and 81% PO selectivity). DFT studies revealed that the interface between NiNPs and Ti^4+^ within the support acts as the active sites responsible for generating peroxide species, thereby facilitating the synthesis of PO. Strong interaction between Ni and Ti takes place during the impregnation, leading to the formation of Ni‐O‐Ti species. With increased Ni loading, the PO selectivity decreases to 14.4%, indicating potential blockage of Ti sites due to excessive Ni. For a cost‐effective comparison, Fe/Ti‐SiO_2_ was also evaluated, demonstrating minimal propylene conversion and complete oxidation of C_3_H_6_ to CO_2_ and water, due to the inherent acidity of Fe that promotes allylic hydrogen abstraction from C_3_H_6_, causing complete oxidation. Li et al. prepared a Ni/TS‐1 catalyst with varying Ni loadings, discovering that a 2% Ni loading yielded a remarkable PO selectivity of 76.8% and a PO formation rate of 151.9 g_PO_ h^−1^ kg^−1^_cat_, surpassing that of conventional Au/TS‐1 under comparable conditions.^[^ ^175^ ^]^ Moreover, the catalyst demonstrated stability for over 20 h. Comprehensive in situ spectroscopic analysis and DFT calculations revealed that NiNPs facilitated the interaction between H_2_ and O_2_, which undergo adsorption on the Ni (111) surface, leading to the creation of mobile O* and OH* species (Figure 14). Following this, O* binds to the hollow site, initiating the formation of a passivation film. As this process continues, the Ni(111) surface becomes quickly covered by the oxygen passivation layer. Subsequently adsorbed O_2_* species interact with the relatively unbound H* to generate intermediate OOH*, leading to the on‐site synthesis of H_2_O_2_. The formed H_2_O_2_ further reacts with propylene to form PO. In addition to these studies, Ni has also shown remarkable promotional effects in ethylene epoxidation when used as a dopant on Ag surface,^[^ ^176^ ^]^ further highlighting its potential as a cost‐effective and high‐performance alternative to gold in selective epoxidation catalysis.

## 6 Efforts at the Macroscale: Reactor Engineering

The process of HOPO requires co‐feeding a mixture of propylene, hydrogen, and oxygen over a catalyst bed. The flammability limits for C_3_H_6_ and H_2_ in the presence of O_2_ are between 2.0% to 59.0% and 4.0% to 95.2%, respectively.^[^ ^177^, ^178^ ^]^ To ensure safety, inert gases like helium, argon, and nitrogen are commonly employed as diluents in a lab‐scale reactor. The meticulous design and proper operation of the reactor are both crucial factors in determining the overall process performance.

**Figure 15:**
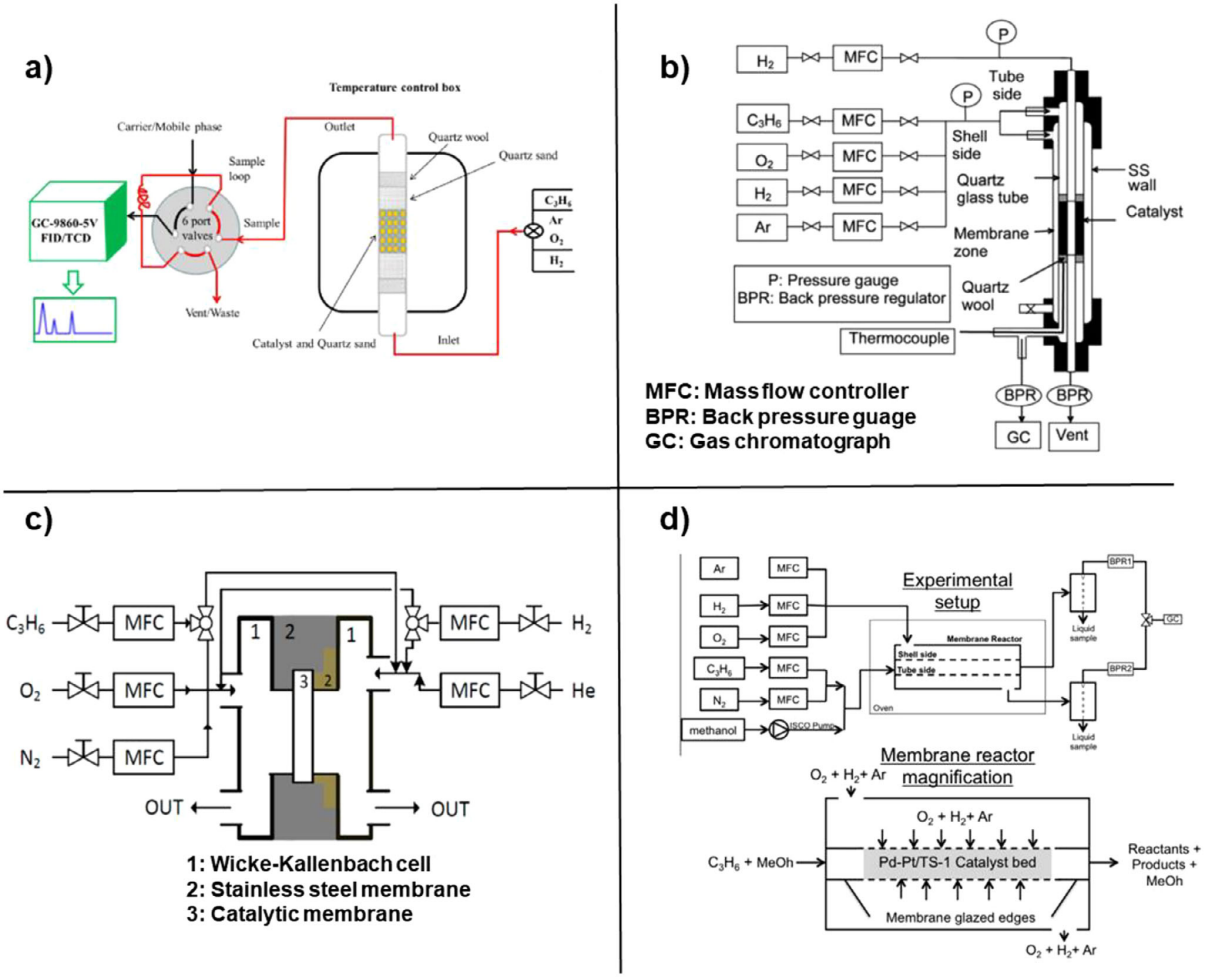
Schematic diagram of several reactor configurations used in lab‐scale studies. a) Conventional packed bed reactor set‐up. Reprinted with the permission from ref. [149] Copyright 2018, Elsevier. b) Catalytic membrane reactor. Reprinted with the permission from ref. [180] Copyright 2008, Elsevier. c) Wicke‐Kallenbach membrane reactor. Reprinted with permission from ref. [182] Copyright 2014, American Chemical Society. d) Continuous flow packed bed membrane reactor. Reprinted with the permission from ref. [183] Copyright 2017, Elsevier.

A packed bed reactor is the most commonly used reactor type in the literature for evaluating the performance of PO catalysts.^[^ ^35^, ^168^ ^]^ In the majority of investigations, a standard reaction feed comprising C_3_H_6_/H_2_/O_2_/inert with volumetric percentages of 10/10/10/70% is employed, positioned safely beyond the flammability limits. The concentrations of the resulting effluents are determined via gas chromatography (GC) analysis. The schematic diagram of a typical reactor set‐up is illustrated in Figure 15a. Harris et al. investigated the kinetics of propylene epoxidation over Au/TS‐1 catalysts using a continuous stirred tank reactor (CSTR), with a focus on the role of product inhibition by PO.^[^ ^17^ ^]^ This study suggested that PO significantly and reversibly inhibits the epoxidation reaction by adsorbing onto the catalytically active sites. Quantitative kinetic analysis reveals a negative reaction order for PO (−0.6 ± 0.2), indicating substantial surface coverage of PO at both Au and Au‐Ti interfacial sites. The inhibition was specific to PO, as neither CO_2_ nor H_2_O affected the reaction rate. It is also reversible, unlike the irreversible deactivation caused by polymeric byproducts. This inhibition alters the kinetics even at low conversions, highlighting the importance of using CSTRs or correction methods for accurate mechanistic insights. A revised two‐site mechanism, accounting for PO adsorption on both sites involved in epoxidation, aligns with the experimental data and ruled out sequential mechanisms.

Sun et al. investigated the influence of particle size on gas phase epoxidation using a resolved particle 3D computational fluid dynamics (CFD) technique.^[^ ^179^ ^]^ They examined three distinct resolved particle models featuring varying particle sizes (6 mm, 8 mm, and 10 mm, referred to as PPD‐6, PPD‐8, and PPD‐10, respectively), allowing for a comprehensive analysis of pertinent aspects such as hot spots, species distribution, and heat transfer rates. These factors are essential for the selection and design of fixed‐bed reactors. The smallest particle size (6 mm) exhibited a shorter diffusion path, promoting quicker diffusion of the product, higher flow velocity, improved radial heat transfer, and a more uniform reaction distribution, resulting in stable flow. However, larger particle packing led to nonuniform flow characterized by pronounced wall effects and deteriorated effective radial mixing of species. Additionally, with increasing particle diameter, the efficiency of heat transfer gradually diminished, resulting in higher hot spot temperatures. Specifically, the PPD‐6 and PPD‐8 models exhibited maximum hot spot temperatures of 245 °C and 249 °C, respectively, while the PPD‐10 model reached 254 °C, the highest among the three. The PPD‐6, PPD‐8, and PPD‐10 models exhibited C_3_H_6_ conversions of 6.2%, 6.0%, and 5.5%, respectively, alongside a corresponding PO selectivity of 92.0%, 91.7%, and 91.4%, respectively.

The PO formation rate can be increased by increasing the partial pressures of H_2_ and O_2_. However, due to safety concerns tied to explosion risks, augmenting reactant concentrations is not a feasible approach within conventional packed bed reactors. Considering this challenge, Oyama and coworkers designed a packed bed catalytic membrane reactor (CMR) (Figure 15b), which allowed them to elevate the reactant concentration from the conventional 10 H_2_/10 O_2_/10 C_3_H_6_/70 Ar ratio to 40 H_2_/40 O_2_/10 C_3_H_6_/10 Ar, which is within the explosive range.^[^ ^180^ ^]^ Structurally, the CMR adopted a concentric tubular layout, wherein the inner tube accommodated the membrane, while the packed bed was positioned within the annular region delineated between the membrane tube and a quartz sleeve. The permeation of H_2_ occurred through the membrane from the tube side, while O_2_ and C_3_H_6_ were introduced from the shell side of the reactor, as shown in Figure 15b. This arrangement facilitated the interaction and subsequent reaction of the reactants within the catalyst bed. This CMR design yielded a notable 100–200% enhancement in the PO production rate when compared to the traditional packed bed reactor, employing an Au/TS‐1 catalyst (Au loading 0.02 wt.%). Sasidharan et al. developed a tubular porous α‐alumina material coated with an Au/TS‐1 membrane on its external surface and assessed its performance within a membrane reactor configuration.^[^ ^181^ ^]^ In this setup, the reactants, 3.1 vol% C_3_H_6_ and 10.2 vol% O_2_, were mixed with 86.7 vol% He and introduced through the α‐alumina tubular core, while 10.6 vol% H_2_ mixed with 86.7 vol% He was fed from an external inlet, enveloping the outer surface of the α‐alumina tubular membrane. As the reactants diffused through the membranes, they interacted with active sites within the membrane structure, initiating the epoxidation reaction. The Au/TS‐1 membrane, with a gold loading of 1.3 wt.%, demonstrated a propylene conversion of 0.29% and a PO selectivity of 89% at 130 °C and 1.5 bar.

Kertalli et al. fine‐tuned the configuration of a CMR by implementing various feeding strategies, and the highest H_2_ efficiency was achieved by introducing C_3_H_6_ and O_2_ separately from H_2_ (Figure 15c).^[^ ^182^ ^]^ This improved performance can be attributed to the high C_3_H_6_ concentrations and restricted H_2_ content within the system. The hydroperoxy species generated on AuNPs are primarily utilized by C_3_H_6_ for the conversion to PO, rather than undergoing hydrogenation to produce water, thus leading to an enhanced H_2_ efficiency. In another study, a Pd‐Pt/TS‐1 catalyst was incorporated into a tubular alumina membrane and evaluated within a packed bed membrane reactor (Figure 15d).^[^ ^183^ ^]^ Methanol, predissolved with C_3_H_6_, was also introduced into the reaction to ensure full catalyst wettability, which is a strategic approach aimed at reducing water formation, thus potentially increasing PO selectivity and productivity. Hölderich and co‐workers also utilized Pd/TS‐1 and Pt‐Pd/TS‐1 catalysts and their performance was assessed in a batch reactor.^[^ ^184^ ^]^ In this setup, liquid C_3_H_6_ was introduced into the reactor vessel along with methanol, followed by the sequential addition of H_2_ (7 bar), N_2_ (15 bar), and O_2_ (10 bar). This study revealed that incorporating Pt (up to 0.02 wt.%) enhanced both PO yield and selectivity by increasing the fraction of Pd (II) species, while excessive Pt loading led to the undesirable hydrogenation of propylene to propane.

To explore catalyst performance within the explosive regime, Nijhuis and co‐workers employed a microreactor system, harnessing its inherent benefits of a small volume with no risk of explosion, and precise temperature control to avert runaway within the system.^[^ ^182^, ^185^ ^]^ The microreactor used in this study consisted of a stainless steel capillary tube with an inner diameter of 0.9 mm. Kinetic studies, leading to proposed rate expressions, were conducted by utilizing a Au/TiO_2_‐SiO_2_ catalyst (with 1 wt.% Au loading) in experiments where feed concentrations ranged across different levels: from 2 to 80 vol% for both H_2_ and O_2_, and from 2 to 40 vol% for C_3_H_6_, while the remaining proportion consisted of helium. Findings from these experiments indicated that increasing H_2_ concentration corresponded to increased epoxidation rates and reduced deactivation, conversely, higher O_2_ concentrations were linked to a decrease in the deactivation rate and facilitates catalyst reactivation. Additionally, augmenting propylene concentration exhibited minimal impact on the PO formation rate but notably reduced the deactivation rate.

## 7 Summary and Outlook

In conclusion, over the last 28 years, the hydroperoxidation of propylene to propylene oxide using H_2_ and O_2_ has witnessed remarkable advancements across various scales, from *nanoscale* to *macroscale*, driven by the pursuit of greener alternative and more efficient catalytic processes. This process follows the dual mechanism where Au, when in close proximity with Ti(IV) sites, enables efficient electron transfer to favor in situ generation of H_2_O_2_ and its subsequent use in epoxidation of propylene. Therefore, the extensive utilization of various types of Ti(IV)‐supported gold catalysts for this reaction has been highlighted, showcasing their catalytic ability and versatility. Calcined or uncalcined TS‐1 has emerged as the most stable and effective support as well as an integral part of the catalytic cycle.^[^ ^69^ ^]^ The precise control of AuNP size can be achieved by manipulating organic‐bound ligands like triphenylphosphine and dodecylthiol, along with pH adjustments and stirring rate variations during the preparation process. The incorporation of promoters like caesium, sulphur, and sodium has significantly elevated catalyst performance through preserving Au and Ti active sites in close proximity, maintaining AuNPs size, and enhancing Au uptake efficiency.^[^ ^114^ ^]^ The catalytic behavior of these AuNPs is found to be intricately tied to their size, with smaller nanoparticles exhibiting higher activity and selectivity. Moreover, the integration of bimetallic nanoparticles, such as Au‐Pd, Au‐Ag, and Au‐Zr, has demonstrated synergistic effects that enhance catalytic activity and stability. An interesting recent discovery is that nickel exhibits notable activity and selectivity in producing PO, showcasing its potential as a cost‐effective alternative to gold.^[^ ^174^ ^]^ This development has expanded the horizons for future research opportunities in this area and suggests the potential for exploring a wider range of metal catalysts in this field.^[^ ^186^ ^]^ Reactor engineering, especially with the incorporation of microreactors and membrane reactors, allowing for process intensification, has enabled the exploration of kinetic regimes previously deemed too risky, providing a deeper understanding of catalytic behavior and reaction mechanism within the explosive regions.

While substantial progress has been achieved in this field, there remain several areas that require further improvements and optimizations as described below, as a first step toward commercialization:

1. The widely employed DP method used to deposit metallic nanoparticles onto zeolitic supports reveals certain limitations, as the method's efficacy is influenced by factors such as the type of precipitating agent, temperature, pH, and stirring rate. Alternative techniques like direct immobilisation of presynthesized NPs hold potential for generating atomically precise nanoparticles and can be applicable across various support materials, including carbonaceous substrates, such as activated carbon, carbon nanotubes and graphene, as well as noncarbonaceous supports like SBA‐15, SiO_2_, TS‐1, Al_2_O_3_, Fe_3_O_4_, ZSM‐5, MCM‐41 and TiO_2_. Titanium can be introduced onto the titania‐free support through a grafting process prior to the deposition.^[^ ^82^ ^]^ The use of various stabilizing ligands, including phosphine, thiol, and amine‐based ligands during nanoparticle synthesis, presents a promising direction to enhance both catalyst stability and selectivity in this domain.
2. Studies on long‐term catalyst stability and recyclability need further expansion. Catalyst stability and recyclability are crucial for any industrial process, as it saves time and resources. Au/Ti‐containing catalyst tends to experience notable deactivation.^[^ ^101^, ^187^ ^]^ As a result, it is important to conduct comprehensive assessments of long‐term stability over extended periods, accompanied by recycling tests spanning multiple cycles. This will help in the optimization and inform strategies to enhance catalyst longevity and sustainability.
3. The incorporation of secondary metals, like silver, copper, zirconium, or nickel to create bimetallic or even trimetallic nanoparticles presents a promising avenue for reducing particle size, preventing nanoparticle aggregation, and potentially enhancing catalytic performance simultaneously.^[^ ^188^ ^]^ However, the exploration of multi‐metallic nanoparticles in the context of HOPO remains relatively limited. Consequently, additional research, including DFT studies and experimentation would be useful in bringing the next generation of high‐output catalysts.
4. While the majority of research has been dedicated to catalyst advancement, the significance of reactor engineering cannot be underestimated.^[^ ^182^ ^]^ Conventional fixed bed reactor setups necessitate a specific ratio of feed concentrations (C_3_H_6_/H_2_/O_2_) to ensure safe operation outside the explosive range. Beyond this, other guiding principles, such as heat management through enthalpy control, downstream separation efficiency, and by‐product handling, must be considered to make the process industrially viable. For instance, the exothermic nature of the reaction requires effective thermal regulation to avoid hot spots and maintain catalyst stability. The cost and purity of feed gases, particularly hydrogen and oxygen, also play a critical role in process economics. In addition, the separation of unreacted gases and management of by‐products like water or oligomeric species add further complexity. Exploring alternative reactor designs, such as membrane reactors or microreactors, could address several of these issues by enabling better control over reactant dosing, heat distribution, and overall process intensification. Further research into the reactor design and configuration will be beneficial to study the reaction kinetics of this process.
5. Computational studies^[^ ^3^, ^189^ ^]^ provide valuable insights into catalyst behavior and the intricacies of the reaction mechanism. The expansion of computational studies is imperative, serving as a guiding tool for catalyst design and optimization. DFT can be effectively employed to study the electron transfer mechanisms occurring between the metal nanoparticles and the supporting materials. Furthermore, it enables an in‐depth exploration of how reactants exhibit preferences for adsorption at distinct surface sites on the catalysts as well as the interactions of promoters with the catalyst surface. Additionally, computational studies can help to elucidate the reaction pathways leading to the formation of PO, aiding in understanding the regioselectivity of epoxide formation. Recent advancements in artificial intelligence (AI)^[^ ^190^, ^191^ ^]^ and machine learning (ML) further complement these approaches by enabling the rapid screening of catalyst candidates, predicting reaction outcomes, developing fundamental models for reaction engineering and optimizing complex reaction networks.

Thus, this comprehensive review highlights the multifaceted advancement in the field of propylene epoxidation, emphasizing on nanoparticles, support structure, and reactor engineering. A holistic perspective, integrating catalyst and reactor design, is recommended. More mechanistic insights are essential and the absence of detailed kinetics for such an import process is remarkable. This understanding will facilitate progress in the catalyst performance, including stability and selectivity, and foster innovation in reactor engineering, addressing challenges across various scales. Only then can this promising process for the greener production of PO be reliably considered for commercialization.

## Conflict of Interest

The authors declare no conflict of interest.

## Data Availability

Data sharing is not applicable to this article as no new data were created or analyzed in this study.
